# Wild edible plants and their cultural significance among the Zhuang ethnic group in Fangchenggang, Guangxi, China

**DOI:** 10.1186/s13002-023-00623-2

**Published:** 2023-11-08

**Authors:** Sizhao Liu, Xinyi Huang, Zhenjun Bin, Bingning Yu, Zushuang Lu, Renchuan Hu, Chunlin Long

**Affiliations:** 1 School of Ethnology and Sociology, Minzu University of China, Beijing, 100081 China; 2 Guangxi Subtropical Crops Research Institute, Nanning, 530010 China; 3 Key Laboratory of Ecology and Environment in Minority Areas (Minzu University of China), National Ethnic Affairs Commission, Beijing, 100081 China; 4 Key Laboratory of Ethnomedicine (Minzu University of China), Ministry of Education, Beijing, 100081 China; 5 College of Life and Environmental Sciences, Minzu University of China, Beijing, 100081 China; 6 Guangxi Institute of Chinese Medicine and Pharmaceutical Science, Nanning, 530022 China; 7 Guangxi Key Laboratory of Traditional Chinese Medicine Quality Standards (Guangxi Institute of Chinese Medicine and Pharmaceutical Science), Nanning, 530022 China; 8 Institute of National Security Studies, Minzu University of China, Beijing, 100081 China

**Keywords:** Wild edible plants, Zhuang, Fangchenggang, Ethnobotany, Traditional knowledge

## Abstract

**Introduction:**

Fangchenggang is situated in the Guangxi Zhuang Autonomous Region, China, renowned for its rich biodiversity and ethnically diverse population. The Zhuang people, constituting the largest minority group in the area, possess a wealth of traditional knowledge concerning wild edible plants (WEPs) owing to the region's favorable environment and dietary customs. With the rapid development of urbanization, tourism, and trade, the Zhuang people's food culture, including the consumption of wild edible plants, has become an attractive aspect of urban development. However, there is almost no comprehensive report available on WEPs consumed by the Zhuang people. The objectives of this study were to: (1) conduct a comprehensive ethnobotanical investigation of the WEPs among the Zhuang people in the region; (2) evaluate the cultural food significance index (CFSI) for the local communities; (3) summarize the cultural characteristics of the wild edible plants consumed, providing scientific support for the development of Fangchenggang as a sustainable and attractive tourism destination.

**Methods:**

Ethnobotanical investigation including market surveys, semi-structured interviews, key informant interviews and participatory observations was conducted in Fangchenggang from January 2021 to March 2023. A total of 137 informants were selected using the snowball method. Information about WEPs, including vernacular names, food categories, parts used, mode of consumption, collecting season, and recipes, was collected and recorded. The CFSI (cultural food significance index) was calculated to identify the most culturally significant WEPs.

**Results:**

A total of 163 species of wild edible plants consumed by the Zhuang people were identified, belonging to 67 families. The main categories of WEPs include wild vegetables (69) and tea substitutes (42). The most commonly consumed parts are fruits (37), followed by whole plants (33) and leaves (21), with herbaceous plants (74) being the most numerous. The availability of wild edible plants remains high throughout the year, with the peak seasons occurring in August and October, and significant abundance also noted in July and November. In the highly significant category (CFSI > 500), a total of 15 plant species were identified, which play a crucial role in the local diet. Additionally, 17 alien species have become part of the local consumption of wild plants, with 7 species listed as invasive alien species.

**Discussion and Conclusions:**

This study documented 163 wild edible plant species and their associated traditional knowledge of the Zhuang people. The research identified culturally significant WEPs and analyzed their multiple uses. The historical development of wild plant consumption in Fangchenggang showed the strong influence of natural and social environments on the Zhuang ethnic group's dietary traditions. The WEPs are characterized by “sour food”, “fresh ingredients” and “cold dishes”, aligning with their health-oriented philosophy of “homology of medicine and food”. Future prospects encompass the cultivation of economically sustainable wild edible plants (WEPs), the preservation of their traits through cross-breeding, ensuring safe consumption through research and safety evaluations, and advocating for the preservation of WEPs' culinary culture to support tourism and sustainable urban development.

## Introduction

Wild edible plants (WEPs) refer to species that are not cultivated and domesticated but are collected from the natural environment and used as food sources [1–3]. For millennia, wild plants have played an essential nutritional role in human survival. Meanwhile this wild source has continued even after the emergence of agriculture and animal husbandry. Many ethnobotanical researches have resulted in the importance of wild edible plants in saving people during famine, drought and war in different developing and developed world countries [4–6].

China is classified among the countries with the richest plant biodiversity in the world. It has a wide variety of wild edible plants (WEPs) with abundant reserves and wide distribution [7]. Several recent studies have described the status of our current knowledge of the use of wild edible plants in China, including the Naxi (168 species), Hani (173 species), Mongolian (90 species), Xizang (90 species), Yi (105 species), and Dulong (148 species) [2, 8–15]. These studies have documented the utilization of local wild edible plants and their associated traditional knowledge. They have used quantitative methods, such as the cultural food significance index and use value to assess their importance within the region. The findings from these investigations hold significant implications, as they contribute not only to the preservation of the traditional knowledge of local wild edible plants but also to the promotion of their sustainable utilization, potentially enhancing the economic income of local residents.

Fangchenggang is situated at the southern tip of of mainland China's coastline, within the Guangxi Zhuang Autonomous Region [16]. The region features mountainous terrain and picturesque sea views. The population comprises four predominant linguistic groups, namely the Han, Zhuang, Yao, and Jing people. The Zhuang ethnic group is the largest minority population [17]. Through extensive interaction with their living environment, the Zhuang people have not only consumed a diverse array of wild edible plants (WEPs) but also acquired valuable traditional ecological knowledge about these plants, which has been shaped by the region's unique topography, unpredictable weather conditions, challenging transportation, and abundant natural resources. With the rapid development of urbanization, tourism, and trade [18], the Zhuang people's food culture, including the consumption of wild edible plants, has become an attractive aspect of urban development.

However, there have been no reports on research related to wild edible plants (WEPs) of the Zhuang people in Fangchenggang, and no comprehensive or quantitative research has been conducted on this topic. Although WEPs were once a primary source of food for the population, most Zhuang people in Fangchenggang have now reduced their consumption. This is due to various factors such as population growth, urbanization, land use changes, and dietary transitions.

Therefore, an ethnobotanical study on WEPs from the Zhuang communities in Fangchenggang is necessary. The objectives of this study are to: (1) conduct a comprehensive ethnobotanical investigation of the WEPs among the Zhuang people in the region; (2) evaluate the cultural food significance index (CFSI) for the local communities; and (3) summarize the cultural characteristics of the wild edible plants consumed, providing scientific support for the development of Fangchenggang as a sustainable and attractive tourism destination.

### Study area

Located in the southern part of Guangxi Zhuang Autonomous Region, Fangchenggang is one of the coastal cities in China that also shares a border with another country [19]. It is also the only city in China with connections to ASEAN (Association of Southeast Asian Nations) countries via sea, land, and river transportation routes. Fangchenggang is located between 20° 36′ N–22° 22′ N latitude and 107° 28′ E–108° 36′ E longitude. It is bordered by Yongning County of Nanning City and Fusui County of Chongzuo City to the north, adjacent to Qinzhou City to the east, Ningming County to the west and faces the Beibu Gulf to the south. Its southwestern border is with the Socialist Republic of Vietnam [20].

Fangchenggang has a diverse geography, featuring mountains and sea. Its terrain is composed of three types: mountains, hills, and coastal mudflats. The region is characterized by the presence of “Three Peninsulas and Three Bays,” including Qisha, Yulian, and Jiangshan peninsulas, as well as Dongwan, Xiwang, and Zhongzhu bays. These features contribute to 100 miles of golden beaches and over 10,000 square kilometers of maritime territory [21, 22]. The main mountain range in the region is the Shiwandashan Mountains (or Shiwan Mountains) and its branches. The Shiwan Mountains run across Fangchenggang City for over 130 km. The highest peak, Shuliangling, is located in Nanping Yao Autonomous Township in Shangsi County, reaching an altitude of 1,462 m above sea level. Apart from the Shiwan Mountains, there are also the Dongshan Mountains, Sifangling Mountains in Shangsi, and Fenghuang Mountains.

The ecological resources in Fangchenggang are abundant. The city experiences ample sunlight, receives abundant rainfall, benefits from favorable hydrothermal conditions, and supports a diverse range of ecosystems, all of which contribute to the presence of a large number of plant species. As of the end of 2020, there were over 2500 wild vascular plant species in Fangchenggang, with three of them being designated as national first-class protected wild plants, including *Hopea chinensis*, *Cycas balansae*, and *Bhesa robusta* [23]. Additionally, there are 20 species of wild plants designated as national second-class protected species, such as *Cibotium barometz*, *Brainea insignis*, and *Ceratopteris thalictroides* [23].

### The people

Fangchenggang is a multi-ligual city with Han, Zhuang, Yao, and Jing people as the main inhabitants. It comprises Shangsi County, Dongxing City, Gangkou District, and Fangcheng District. According to the 7th national census data in 2021, the city's permanent population is 1.05 million, of which the Zhuang ethnic group is the largest minority with a population of 355,041, accounting for 33.94% of the total population [24–26].

In recent decades, with the increasing number of people coming to Fangchenggang for business and employment, as well as the further opening up of household registration policies, the number of ethnic minorities moving into Fangchenggang has been on the rise. Ethnic diversity in Fangchenggang fosters a dynamic exchange of knowledge, ongoing interactions, communication, and cultural amalgamation. This has given rise to a distinctive culinary culture intricately linked with the local ecological and social environment of the Zhuang people.

The main staple food of the Zhuang people in the Fangchenggang region is rice, and other cereals such as corn, sweet potatoes, taro, and cassava are used as supplementary foods [27]. For breakfast and lunch, most people eat rice porridge and rice noodles with simple vegetable dishes such as pickled vegetables. Dinner usually consists of rice with meat dishes such as fish and meat. The Zhuang people enjoy sour food, and men usually like to drink alcohol. A significant portion of Zhuang snacks, particularly during festivals and holidays, involves the use of glutinous rice to create a variety of cakes. These delightful treats include zongzi (glutinous rice wrapped in bamboo leaves), deep-fried glutinous rice balls known as oily balls, and sticky rice cakes [28].

## Materials and methods

### Definition of wild edible plants

Following Heywood’ [29] definition of non-cultivated plants as: “plants that grow spontaneously in self-maintaining populations in natural or semi-natural ecosystems and can exist independently of direct human action, we consider as ‘wild’ all plants that are gathered (not cultivated), even if they grow on cultivated rather than uncultivated or forest land.” Further, wild edible plants include plants whose parts can be consumed as vegetables, fruits, spices, nuts, dyes, liquors, oils and fats, snacks and are not exclusively used for treating (self-)diagnosed illnesses [30].

### Field survey and data collection

**Fig. 1:**
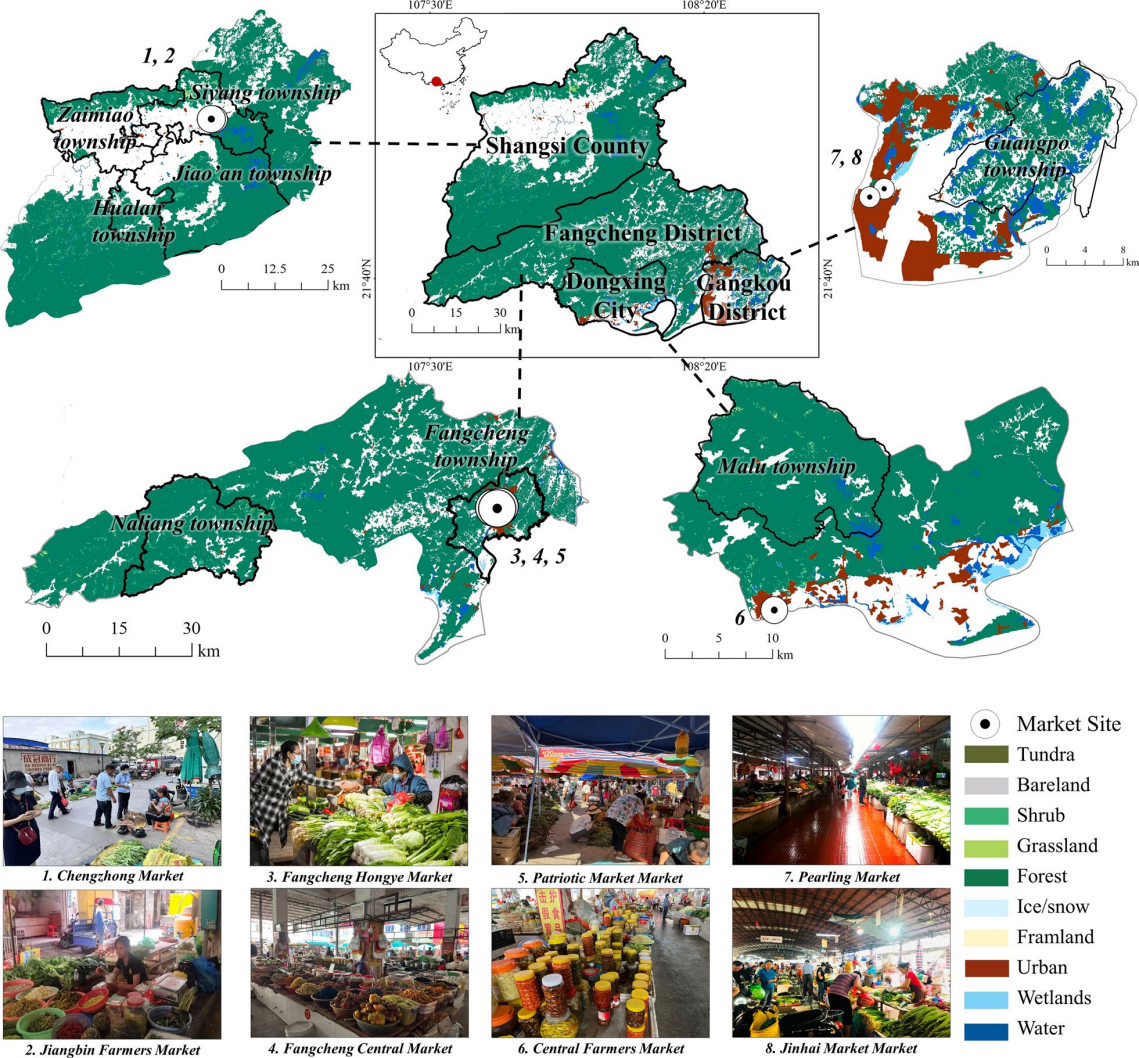
Sketch map of the study area

Ethnobotanical surveys were carried out in Fangchenggang from January 2021 to March 2023 (Fig. 1). Data were gathered in three stages: (1) In the first stage, the researchers visited the main markets within the Zhuang communities in Fangchenggang through the market survey method [31, 32]. A total of 8 markets (Table 1) and some road-side selling points were visited to obtain preliminary information about the wild edible plants commonly consumed and purchased by the people. We selected 69 informants for further interviews, including the origin, local name, availability, use part, processing method, mouthfeel, whether it is used as a medical diet, and other uses; and (2) Based on the information gathered during the market surveys, we applied the snowball sampling method to select a total of 68 informants for interviews from 8 townships (Table 1).

**Table 1:** Study site locations and demographic characteristics of respondents

Shangsi county									Fangcheng district								
Type	Gender		Age			Occupation			Type	Gender		Age			Occupation		
	M	F	< 30	30–60	> 60	Retailer	Farmer	Salary worker		M	F	< 30	30–60	> 60	Retailer	Farmer	Salary worker
*Markets*									*Markets*								
Jiangbin Farmers	7	5	1	9	2	9	0	3	Hongye	3	4	1	5	1	6	0	1
Chengzhong	4	5	1	7	1	8	0	1	Central	5	3	0	6	2	6	1	1
									Patriotic	3	6	2	4	3	7	2	0
*Townships*									*Townships*								
Jiao'an	3	5	1	2	5	1	7	0	Na liang	3	3	0	2	4	1	5	0
Si yang	6	4	1	5	4	0	8	2									
Zai miao	4	5	2	3	4	1	7	1	Fang cheng	6	3	1	3	5	1	7	1
Hua lan	4	5	1	4	4	0	9	0									
*Total*									*Total*								
57	28	29	7	30	20	19	31	7	39	20	19	4	20	15	21	15	3

Dongxing city									Gangkou district								
Type	Gender		Age			Occupation			Type	Gender		Age			Occupation		
	M	F	< 30	30–60	> 60	Retailer	Farmer	Salary worker		M	F	< 30	30–60	> 60	Retailer	Farmer	Salary worker
*Markets*									*Markets*								
Central farmers	2	3	1	4	0	4	1	0	Pearling	4	5	1	6	2	7	1	1
									Jin hai	3	7	1	8	1	7	1	2
*Townships*									*Townships*								
Ma lu	6	4	1	4	5	1	8	1	Guang po	4	3	0	5	2	1	6	0
*Total*									*Total*								
15	8	7	2	8	5	5	9	1	26	11	15	2	19	5	15	8	3

We used a comprehensive approach to data collection, including free listing, semi-structured interviews, key informant interviews, and participatory observation. All wild plants that are still regularly used or have been used in the past, and the following questions were also asked during the interviewing:

1. Is this species very common, common, of medium frequency, or rare?
2. When was the last time that you tried this plant?
3. Which plant parts have you consumed?
4. How do you use and prepare this plant?
5. How much do you appreciate this plant? Please give your score between 1 and 10.
6. Does this plant have any medicinal properties, and if so, which part is used as medicine.

For the identification of plants, the voucher specimens were studied and compared with reference books (*Flora Rpublicae Popularis Sinicae*, *Flora of China*, *Flora of Guangxi*) and electronic online resources (http://www.iplant.cn/ and www.worldfloraonline.org). The nomenclature of all vascular plants follows *Flora of China*. Prof. Chunlin Long and Renchuan Hu identified all plant species, and the voucher specimens were deposited in the herbarium of the Guangxi institute of Chinese Medicine & Pharmaceutical Science, in Nanning.

### Data analysis

The cultural food significance index (CFSI) considers a wide variety of factors in the evaluation of a specific wild edible plant. CFSI was calculated to evaluate the cultural significance of wild edibles using the formula given by Pieroni [33].

$$\begin{aligned}{\text{CFSI}} &={\text{QI}}\, \times \,{\text{AI}}\, \times \,{\text{FUI}} \times \,{\text{PUI}}\\&\quad \times \,{\text{MFFI}} \times \,{\text{TSAI}}\, \times \,{\text{FMRI}}\, \times \,10^{ - 2}\end{aligned}$$

The CFSI includes quotation frequency (QI, frequency of quotation index: the number of people who mentioned a plant among all informants), availability [AI, availability index: divided into very common (4.0), common (3.0), average (2.0) and uncommon (1.0), Correction Index: Widespread (=), In some places (− 0.5), In a particular place (− 0.1)], typology of the used parts [PUI, parts used index: divided into Whole plant (2.0), Leaf (2.0), Root (2.0), Branches and leaves (2.0), Tender branches and leaves (1.5), Fruit (1.50), Rhizome (1.5), Young shoot (1.25), Seed (1.0), Tender stem (1.0), Bark (1.0), Tender leaf (0.75), Flower (0.75), frequency of use (FUI, frequency of utilization index: divided into more than once a week (5.0), once a week (4.0), once a month (3.0), more than once a year but less than once a month (2.0), once a year (1.0) and unused for nearly 30 years (0.5)], kind and a number of food uses [MFFI, multifunctional food use index: divided into raw food and cold salad (1.5), boiling, stewing and seasoning (1.0), special purpose and condiments (0.75) and raw food as snacks (0.50)], taste appreciation [TSAI, taste score appreciation index: divided into excellent (10.0), very good (9.0), good (7.5), fair (6.5), poor (5.5) and very poor (4.0)] and perceived role as food medicine [FMRI, food-medicinal role index: divided into very high (as medicinal food: 5.0), high (as medicine to treat a certain disease: 4.0), moderately high (very healthy food: 3.0), moderately low (healthy food, unknown efficacy: 2.0) and unknown or possibly toxic (1.0)]. The use of this index allows exploring potential wild greens, as they exist in different climatic zones [8].

## Results

### Use of wild edible plants by the Zhuang community

A total of 137 Zhuang respondents were selected for interviews, including 57 from Shangsi County, 39 from Fangcheng District, 15 from Dongxing City, and 26 from Gangkou District. Among these respondents, 67 were male, while 70 were female. The age of the respondents ranged from 17 to 94, with the majority falling within the 30–60 age group (Table 1). The informants had diverse occupations, including farmers, wage workers, and traders, with farmers being the most prominent group. They reported a total of 163 Wild Edible Plants (WEPs), and detailed ethnobotanical information about these plants is provided in Table 2. This information includes the scientific name, vernacular name, Chinese name, family, habit, food category, used part, mode of consumption, collection season, recipe, specimen number, and CFSI.

**Table 2:** List of wild edible plants used by Zhuang

Scientific name	Chinese name	Local name in pipin	Family	Habit	Food categories	Part used	Mode of consumption	Example recipes	Collection season	QI(137)	AI	FUI	PUI	MFFI	TSAI	FMRI	CFSI	Endangered situation	Voucher number
*Abrus precatorius* L	相思子	Hou ta te	Fabaceae	Tree	Ts	Whole plant	Water-boiled	–	9–12,2	34	3.5	3	2	1	9	5	321.30	LC	HRC1320
*Abrus pulchellus* subsp. *mollis* (Hance) Verdc	毛相思子	Jj guo cao, Xiangsicha	Fabaceae	Herb	Wv; Ts	Whole plant	Stew soup; Water-boiled	Pork rib stew soup	9–12,1	73	2	3	2	0.75	10	5	328.50	LC	HRC1355
*Abutilon indicum* (L.) Sweet	磨盘草	Gi mu sei	Malvaceae	Herb	Sn	Seed	Eat directly	–	2–3	12	2.5	1	1	0.5	6.5	4	3.90	LC	HRC1355
*Alpinia oblongifolia* Hayata	华山姜	–	Zingiberaceae	Herb	Wv; Sp	Young shoot,Tuber	Stir-fry	Stir-fried pork with young shoots	1–12	35	3.0	2.0	1.25	1	10	4	126.00	LC	HRC1287
*ernanthera philoxeroides* (Mart.) Griseb. *	空心莲子草	–	Amaranthaceae	Herb	Wv	Leaf	Stir-fry	Stir-fried *Alternanthera philoxeroides*	2–6	11	3.5	2.0	2.00	1	6.5	3	30.03	LC	HRC1337
*Amaranthus spinosus* L. *	刺苋	Ge po hong nan	Amaranthaceae	Herb	Wv; Ts	Whole plant	Stir-fry; Water-boiled	Stir-fried *Amaranthus spinosus*	1–5	25	2.5	2.0	2.00	1	7.5	3	56.25	LC	HRC1477
*Amaranthus tricolor* L. *	苋	Ge po hong	Amaranthaceae	Herb	Wv	Whole plant	Stir-fry; Stew soup	Stir-fried pork with young shoots	1–5	85	2.5	2.0	2.00	1	7.5	3	191.25	LC	HRC1319
*Anoectochilus roxburghii (*Wall.) Lindl	金线兰	Jin xian lian	Orchidaceae	Herb	Ts	Whole plant	Soak in boiling water	–	1–12	60	1.5	3.0	2.00	1	6.5	5	175.50	EN	HRC1332
*Aralia armata* (Wall. ex G.Don) Seem	野楤头	Ci gan	Araliaceae	Shrub	Wv	Young shoot	Cold toss	–	2–3	19	3.0	2.0	1.25	1.5	7.5	3	48.09	LC	HRC1309
*Artemisia argyi* H.Lév. & Vaniot	艾	Ai	Asteraceae	Herb	Wv	Tender leaf; Whole plant	Stir-fry; Water-boiled	Pound and mix glutinous rice flour to make glutinous rice cakes (ciba)	2–3	89	4.0	2.0	0.75	1.5	7.5	5	300.38	LC	HRC1487
*Artemisia indica* Willd	五月艾	Ge ai	Asteraceae	Herb	Wv	Whole plant	Water-boiled; Make filling	Mung bean paste filling glutinous rice balls	2–3	35	3.0	2.0	0.75	1.5	7.5	5	88.59	LC	HRC1316
*Artemisia lactiflora* Wall. ex DC	白苞蒿	Yi mu ai	Asteraceae	Herb	Wv	Whole plant	Water-boiled	Cook together with eggs	2–3	20	3.0	1.0	0.75	1	7.5	5	16.88	LC	HRC1350
*Aster indicus* L	马兰	Ma lan tou	Asteraceae	Herb	Wv	Young shoot	Cold toss	Cold tossed dried tofu	2–3	6	3.5	2.0	1.25	1.5	7.5	4	23.63	LC	HRC1351
*Astragalus sinicus* L	紫云英	Hong hua cao	Fabaceae	Herb	Wv	Tender branches and leaves	Cold toss; Stir-fry; Stew soup	Cold toss	2–3	6	2.0	1.0	0.75	1.5	10	3	4.05	LC	HRC1352
*Asystasia nemorum* Nees	十万错	–	Acanthaceae	Herb	Fd	Branches and leaves	Stir-fry and then Soak in boiling water	Dyeing glutinous rice (purple-red color)	3	57	2.0	1.0	2.00	1.5	7.5	4	102.60	LC	HRC1353
*Bambusa blumeana* Schult.f	簕竹	–	Poaceae	Bamboo	Wv	Tender stem	Stir-fry	Stir-fried with dried meat and chili	2–4	30	3.0	2.0	1.00	1	9	3	48.60	LC	HRC1354
*Bambusa chungii* McClure	粉单竹	–	Poaceae	Bamboo	Wv	Tender stem	Stir-fry	Stir-fried with dried meat and chili	5–10	61	3.5	2.0	1.00	1	9	3	115.29	LC	HRC1359
*Basella alba* L. *	落葵	Fo weng	Basellaceae	Herb	Wv	Leaf	Water-boiled; Stir-fry	Stir-fried	1–12	68	2.5	3.0	2.00	1	7.5	5	382.50	LC	HRC1360
*Bidens pilosa* L. *	鬼针草	Ke mu gong	Asteraceae	Herb	Wv	Tender leaf	Cold toss; Stir-fry; Stew soup; Make filling	Stir-fried with pork belly	2–4	13	4.0	2.0	0.75	1.5	6.5	4	30.42	LC	HRC1281
*Bischofia javanica* Blume	秋枫	Ge mei tong	Phyllanthaceae	Tree	Fd	Bark	Water-boiled and soak	Dyeing glutinous rice (black color)	3	8	2.5	2.0	1.00	1	6.5	3	7.80	LC	HRC1328
*Blechnum orientale* L	乌毛蕨	–	Aspleniaceae	Herb	Wv	Young shoot; Tuber	Stir-fry	Crush the tuber, filter to extract starch and make fern cakes; Stir-fry young shoots	1–12	8	4.0	2.0	1.25	1	6.5	4	20.8	LC	HRC1355
*Buddleja officinalis* Maxim	密蒙花	Ran fan hua	Scrophulariaceae	Shrub	Fd	Flower	Soak in boiling water	Dyeing glutinous rice (yellow color)	3–4	71	3.0	1.0	0.75	1	7.5	4	47.9	LC	HRC1356
*Camellia drupifera* Lour	越南油茶	Ge mei sha	Theaceae	Tree	Of	Fruit	Extract oil	–	10–12	52	3.0	3.0	1.50	1.5	9	4	442.3	LC	HRC1357
*Camellia euphlebia* Merr. ex Sealy	显脉金花茶	–	Theaceae	Shrub or Dungarunga	Of、Ts	Fruit、Flower	Fruit: extract oil; Flower: soak in boiling water after drying	–	2,7	12	1.5	2.0	1.50	1.5	7.5	3	18.2	VU	HRC1318
*Camellia indochinensis* var. *tunghinensis* (Hung T.Chang) T.L.Ming & W.J.Zhang	东兴金花茶	–	Theaceae	Shrub	Of、Ts	Fruit、Flower	Fruit: extract oil; Flower: soak in boiling water after drying	–	1,7	8	1.5	2.0	1.50	1.5	7.5	3	12.2	EN	HRC1338
*Camellia oleifera* C.Abel	油茶	–	Theaceae	Tree	Of	Fruit	Extract oil	–	10–12	106	4.0	5.0	1.50	1.5	9	4	1717.2	LC	HRC1314
*Camellia petelotii* (Merr.) Sealy	金花茶	Deng hua cha	Theaceae	Shrub	Of、Ts	Fruit、Flower	Fruit: extract oil; Flower: soak in boiling water after drying	–	1–2	76	2.0	3.0	1.50	1.5	7.5	3	269.3	VU	HRC1366
*Campanumoea javanica* Bl	金钱豹	–	Campanulaceae	Liana	Wf; Wv	Fruit	Fruit: eat directly; Root: stew soup	Stew with meat (chicken, pork, duck)	10,1–12	65	1.5	3.0	1.50	1	10	3	131.6	LC	HRC1371
*Canarium album* (Lour.) DC	橄榄	Mong gu	Burseraceae	Tree	Sp	Fruit	Eat directly	–	10–12	82	2.5	2.0	1.50	0.5	9	3	83.0	LC	HRC1372
*Canarium pimela* Leenh	乌榄	Mong mei	Burseraceae	Tree	Sp; Wf	Fruit	Marinate	Pickled vegetables for cooking fish or eating congee	10–12	76	2.5	3.0	1.50	0.75	9	3	173.1	LC	HRC1380
*Capsella bursa-pastoris* (L.) Medik	荠	Ma hiu	Brassicaceae	Herb	Wv	Branches and leaves	Cold toss; Stir-fry; Make filling	Pickled vegetables; Pork and shepherd's purse dumplings	2–3	61	2.0	2.0	2.00	1.5	9	4	263.5	LC	HRC1295
*Castanopsis hystrix* Miq	红锥	Dong mei ge deng	Fagaceae	Tree	Wf; Nu	Fruit	Stir-fry	–	8–11	62	2.5	2.0	1.50	1	10	3	139.5	LC	HRC1376
*Centella asiatica* (L.) Urb	积雪草	Ge pa dun	Apiaceae	Herb	Wv; Ts	Whole plant	Stir-fry; Water-boiled; Cold toss	Cold tossed with walnuts	1–12	105	3.0	4.0	2.00	1	7.5	5	945.0	LC	HRC1293
*Choerospondias axillaris* (Roxb.) B. L. Burtt & A. W. Hill	南酸枣	Ge meng ma	Anacardiaceae	Tree	Wf	Fruit	Eat directly	–	10–12	37	2.5	2.0	1.50	0.5	6.5	5	45.1	LC	HRC1381
*Cibotium barometz* (L.) J. Sm	金毛狗	Ge gun wang	Cyatheaceae	Herb	Wv	Young shoot; Tuber	Stir-fry	Crush the tuber, filter to extract starch and make fern cakes; Stir-fry young shoots	1–12	5	3.0	2.0	1.25	1.5	7.5	5	12.7	LC	HRC1383
*Cinnamomum parthenoxylon* (Jack) Meisn	黄樟	Ge mei zhong	Lauraceae	Tree	Of	Tree trunk	Steam	Tree trunk sliced, steamed in a pot, oil distilled from the steam, and the oil used for stir-frying	4–10	8	2.0	2.0	1.00	1.5	9	3	13.0	LC	HRC1291
*Citrus limonia* Osb	黎檬	–	Rutaceae	Tree	Wf; Sp	Fruit	Eat directly	–	5–12	88	3.5	4.0	2.00	0.75	10	5	970.2	LC	HRC1384
*Colocasia esculenta* (L.) Schott	芋	Ge piu	Araceae	Herb	Wv	Petiole	Cold toss	–	1–12	25	2.0	2.0	1.00	1.5	6.5	3	29.3	LC	HRC1386
*Crassocephalum crepidioides* (Benth.) S. Moore *	野茼蒿	Ge pa song hao dui	Asteraceae	Herb	Wv	Whole plant	Stir-fry; Water-boiled	Cooking in hot pot	3–4	25	4.0	1.0	0.75	1	7.5	4	22.5	LC	HRC1389
*Cratoxylum cochinchinense* (Lour.) Blume	黄牛木	Ge mei guo shu	Hypericaceae	Shrub or Dungarunga	Ts	Branches and leaves	Water-boiled	–	1–12	53	3.0	3.0	2.00	1	6.5	5	310.05	LC	HRC1289
*Cryptotaenia japonica* Hassk	鸭儿芹	–	Apiaceae	Herb	Wv	Branches and leaves	Cold toss; Stir-fry	Cold tossed with mushrooms	12, 1–3	52	3.0	2.0	2.00	1.5	7.5	3	210.60	LC	HRC1307
*Curcuma longa* L	姜黄	Ge huang jiong	Zingiberaceae	Herb	Fd	Rhizome	Water-boiled	–	9–12	61	3.0	2.0	1.50	1.5	7.5	5	308.81	LC	HRC1388
*Curcuma phaeocaulis* Valeton	莪术	Ge zha mu gong	Zingiberaceae	Herb	Wv	Rhizome	Stew soup	Stew soup with pork ribs and chicken	9–12	28	2.5	2.0	1.50	1	7.5	5	78.75	LC	HRC1342
*Cyclocodon lancifolius* (Roxburgh) Kurz	轮钟草	–	Campanulaceae	Herb	Wf	Fruit	Eat directly	–	8–12, 1	55	1.5	2.0	1.50	0.5	10	3	37.13	LC	HRC1390
*Dioscorea persimilis* Prain & Burkill	褐苞薯蓣	–	Dioscoreaceae	Liana	Wv	Root	Stir-fry	Stir-fry with preserved meat	11–12, 1–3	81	3.0	2.0	1.50	1	7.5	3	164.03	LC	HRC1286
*Diplopterygium chinense* (Rosenstock) De Vol	中华里白	–	Gleicheniaceae	Herb	Wv	Young shoot	Stir-fry	Stir-fry with preserved meat	1–12	11	3.0	2.0	1.25	1	9	4	29.70	LC	HRC1392
*Eclipta prostrata* (L.) L	鳢肠	Ge ong gai	Asteraceae	Herb	Wv	Tender leaf	Water-boiled; Stir-fry	Cooking with congee	1–3	8	4.0	1.0	0.75	1	6.5	4	6.24	LC	HRC1391
*Elephantopus scaber* L	地胆草	Ge san shi la	Asteraceae	Herb	Wv; Ts	Root、Whole plant	Stew soup; Water-boiled	Cooking together with chicken broth, duck broth	1–12	88	3.5	4.0	2.00	0.75	10	5	970.2	LC	HRC1274
*Embelia laeta* (L.) Mez	酸藤子	Ma song gu	Primulaceae	Liana	Wf	Fruit	Eat directly	–	4–7	18	3.0	2.0	1.50	0.5	7.5	3	29.3	LC	HRC1305
*Embelia ribes* Burm.f	白花酸藤果	–	Primulaceae	Liana	Wf	Fruit	Eat directly	–	4–7	13	2.0	2.0	1.50	0.5	7.5	3	22.5	LC	HRC1392
*Embelia ribes* subsp. *pachyphylla* (Chun ex C. Y. Wu & C. Chen) Pipoly & C. Chen	厚叶白花酸藤果	–	Primulaceae	Liana	Wf	Fruit	Eat directly	–	4–7	11	2.0	2.0	1.50	0.5	7.5	3	808.5	LC	HRC1393
*Emilia sonchifolia* (L.) DC	一点红	Yi dian hong	Asteraceae	Herb	Wv	Tender leaf	Stew soup	Cooking together with chicken broth, duck broth, and bone broth	1–12	76	3.0	3.0	0.75	0.75	9	5	18.2	LC	HRC1394
*Engelhardia roxburghiana* Lindl	黄杞	Ge mei ba hong	Piperaceae	Tree	Ts	Leaf	Water-boiled after dry	–	1–12	42	3.5	3.0	2.00	1	9	5	8.8	LC	HRC1290
*Epaltes australis* Less	球菊	E bu shi cao	Asteraceae	Herb	Ts	Whole plant	Water-boiled	–	1–12	13	3.5	1.0	0.75	1	6.5	5	7.4	LC	HRC1329
*Eryngium foetidum* L. *	刺芹	Yue nan xiang cai	Apiaceae	Herb	Sp	Whole plant	Eat directly	Important ingredients for Dongxing Jing ethnicity's "Qu tou dan"	1–12	65	3.0	4.0	2.00	0.5	9	4	173.1	LC	HRC1250
*Ficus auriculata* Lour	大果榕	Ge mei dei nong	Moraceae	Tree	Wf	Fruit	Eat directly	–	1–12	26	2.0	2.0	1.50	0.5	6.5	3	396.9	LC	HRC1270
*Ficus hirta* Vahl	粗叶榕	Ge mei nong gei	Moraceae	Shrub or Dungarunga	Wf; Wv	Fruit; Root	Stew soup	–	5–8	69	3.0	3.0	1.50	0.5	9	5	11.1	LC	HRC1395
*Ficus microcarpa* L. f	榕树	Ge mei nong xie	Moraceae	Tree	Wv	Root	Stew soup	Stew soup with pork backbone	1–12	6	3.0	2.0	1.50	0.75	7.5	5	280.8	LC	HRC1331
*Ficus oligodon* Miq	苹果榕	–	Moraceae	Tree	Wf	Fruit	Eat directly	–	5–6	18	2.0	2.0	1.50	0.5	7.5	3	15.2	LC	HRC1325
*Ficus pumila* L	薜荔	Liang fen guo	Moraceae	Shrub	Sn	Fruit	Pound	Chilled noodles	5–8	39	2.5	2.0	1.50	1	9	3	244.5	LC	HRC1346
*Garcinia multiflora* Champ. ex Benth	木竹子	–	Clusiaceae	Tree	Wf	Fruit	Eat directly	–	9–12	28	2.0	2.0	1.50	0.5	6.5	3	16.38	LC	HRC1302
*Garcinia oblongifolia* Champ. ex Benth	岭南山竹子	Ge mei lu dong	Clusiaceae	Tree	Wf	Fruit	Eat directly	–	9–12	30	2.5	2.0	1.50	0.5	6.5	3	21.94	LC	HRC1399
*Gardenia jasminoides* J.Ellis	栀子	Ge mei leng	Rubiaceae	Shrub	Wv; Fd	Flower,Fruit	Cold toss;Fry; Soak in water after drying	Dyeing glutinous rice (yellow color)	3–7, 5–12	43	3.5	2.0	1.50	1.5	6.5	5	220.11	LC	HRC1396
*Glycosmis pentaphylla* (Retz.) DC	山小橘	Ma long liu	Ulmaceae	Shrub or Dungarunga	Wf	Fruit	Eat directly	–	11–12, 1–3	23	2.5	2.0	1.50	0.5	7.5	3	19.41	LC	HRC1397
*Gomphandra tetrandra* (Wall.) Sleumer	粗丝木	Shan luo bo	Stemonuraceae	Shrub or Dungarunga	Ts	Root	Water-boiled	–	1–12	9	2.0	2.0	1.50	1	7.5	5	20.25	LC	HRC1398
*Grona styracifolia* (Osbeck) H. Ohashi & K. Ohashi	广东金钱草	Jin qian cao	Fabaceae	Herb	Ts	Whole plant	Water-boiled	–	8–11	82	2.5	4.0	2.00	1	9	5	645.8	LC	HRC1279
*Gynostemma pentaphyllum* (Thunb.) Makino	绞股蓝	–	Piperaceae	Herb	Ts	Whole plant	Soak in boiling water	–	8–12	105	2.5	3.0	2.00	1	9	5	708.8	LC	HRC1402
*Gynura divaricata* (L.) DC	白子菜	Ming yue cao	Asteraceae	Herb	Wv	Tender leaf	Stir-fry; Water-boiled	Stir-fry with preserved meat	2–5	20	2.0	2.0	0.75	1	6.5	4	15.6	LC	HRC1403
*Habenaria dentata* (Sw.) Schltr	鹅毛玉凤花	–	Orchidaceae	Herb	Wv	Tuber	Stew soup	Stew with fish	8–12	18	1.5	2.0	1.50	1	6.5	5	26.3	LC	HRC1343
*Hedyotis effusa* Hance	鼎湖耳草	Long kou gan	Rubiaceae	Herb	Ts	Whole plant	Water-boiled	–	1–12	51	2.5	2.0	2.00	1	6.5	5	248.6	LC	HRC1406
*Helixanthera parasitica* Lour	离瓣寄生	Sang ji sheng	Loranthaceae	Shrub	Ts	Branches and leaves; Stem	Water-boiled	–	1–12	15	2.0	2.0	2.00	1	6.5	5	39.0	LC	HRC1407
*Hellenia speciosa* (J.Koenig) S.R.Dutta	闭鞘姜	–	Costaceae	Herb	Wv	Tender stem	Stir-fry; Stew soup	Pan-fry first, then stew with pig's head meat	1–12	21	2.0	2.0	1.50	1	9	4	45.4	LC	HRC1345
*Houttuynia cordata* Thunb	蕺菜	Yu xing cao	Saururaceae	Herb	Wv; Sp	Whole plant	Stir-fry; Water-boiled; Cold toss	The stems and leaves are used for a light stir-fry; the roots are used for cold tossing, and as a dipping sauce for raw fish	1–12	93	3.0	4.0	2.00	1	7.5	5	732.4	LC	HRC1294
*Hypericum japonicum* Thunb	地耳草	Tian ji huang	Hypericaceae	Herb	Ts	Whole plant	Water-boiled	–	4–12	68	3.5	3.0	2.00	1	6.5	5	464.1	LC	HRC1275
*Ilex confertiflora* Merr	密花冬青	Qing ming cha	Aquifoliaceae	Shrub or Dungarunga	Ts	Young shoot	Soak in boiling water	–	1–12	71	1.0	1.0	0.75	1	9	5	23.96	LC	HRC1408
*Imperata cylindrica* (L.) Raeusch	白茅	Ge ha wai	Poaceae	Herb	Wv; Ts	Root; Young shoot	Stir-fry; Water-boiled	–	1–12	83	4.0	3.0	1.50	1	7.5	5	560.3	LC	HRC1261
*Isodon serra* (Maxim.) Kudô	溪黄草	Kui huang cao	Lamiaceae	Herb	Ts	Whole plant	Stew soup	Stew with grass carp	4–8	63	1.5	2.0	2.00	1	6.5	4	147.4	LC	HRC1317
*Juncus effusus* L	灯芯草	–	Juncaceae	Herb	Ts	Whole plant	Water-boiled	–	1–12	27	2.5	2.0	2.00	1	6.5	5	87.8	LC	HRC1409
*Leonurus japonicus* Houttuyn	益母草	–	Lamiaceae	Herb	Wv; Ts	Whole plant	Stew soup; Stir-fry	Stew soup withchicken	2–5, 6–11	43	3.4	2.0	2.00	1	7.5	5	219.3	LC	HRC1312
*Ligustrum lucidum* W.T.Aiton	女贞	–	Oleaceae	Tree	Lb	Fruit	Brewing	–	8–12	26	3.0	2.0	1.50	1	6.5	5	76.1	LC	HRC1324
*Liquidambar formosana* Hance	枫香树	Ge mei lou	Altingiaceae	Tree	Fd	Leaf	Soak in water after drying	Dyeing glutinous rice	3–4	109	3.0	1.0	2.00	1.5	9	4	353.2	LC	HRC1252
*Lithocarpus litseifolius* (Hance) Chun	木姜叶柯	Tian cha	Fagaceae	Tree	Ts	Leaf	Soak in water after drying	–	1–12	45	1.5	3.0	2.00	1	10	4	162.0	LC	HRC1300
*Lithocarpus pachylepis* A. Camus	厚鳞柯	Feng liu guo	Fagaceae	Tree	Nu	Fruit	Brewing	–	10–12	25	1.5	2.0	1.50	0.5	6.5	3	11.0	VU	HRC1410
*Litsea cubeba* (Lour.) Pers	山鸡椒	–	Lauraceae	Tree	Sp	Fruit	Water-boiled	–	2–11	73	3.0	3.0	1.50	1	9	5	591.3	LC	HRC1264
*Litsea pungens* Hemsl	木姜子	Mei zhong heng	Lauraceae	Tree	Sp	Leaf	Stir-fry	Stir-fry with meat	6–8	20	2.5	2.0	1.50	1	10	5	75.0	LC	HRC1301
*Lonicera confusa* DC	华南忍冬	Ge gen wa gen ang	Caprifoliaceae	Liana	Ts	Whole plant	Soak in boiling water	–	1–12	31	1.5	2.0	0.75	1	5.5	5	19.2	LC	HRC1411
*Lonicera macrantha* (D. Don) Spreng	大花忍冬	Ge gen wa gen ang	Caprifoliaceae	Liana	Ts	Whole plant	Soak in boiling water	–	1–12	25	1.5	2.0	0.75	1	5.5	5	15.5	LC	HRC1271
*Lophatherum gracile* Brongn	淡竹叶	Ge ma an gong	Poaceae	Herb	Ts	Whole plant	Water-boiled	–	1–12	92	3.5	3.0	2.00	1	7.5	5	724.5	LC	HRC1273
*Lycium chinense* Mill	枸杞	E gou ji	Solanaceae	Shrub	Wv; Lb	Branches and leaves,Fruit	Water-boiled; Brewing	Cook in hot pot, dry pot, or porridge	1–12, 6–12	23	1.5	3.0	1.50	1	9	5	81.5	LC	HRC1277
*Macrosolen cochinchinensis* (Lour.) Tiegh	鞘花	–	Loranthaceae	Shrub	Ts; Wf	Leaf; Fruit	Water-boiled	–	1–12, 5–8	26	2.5	2.0	1.50	1	6.5	5	63.4	LC	HRC1327
*Magnolia figo* (Lour.) DC	含笑花	–	Magnoliaceae	Shrub	Ts	Flower	Soak in boiling water	–	3–5	6	2.0	1.0	0.75	1	9	3	2.4	LC	HRC1283
*Melastoma malabathricum* L	印度野牡丹	Ge mei nan	Melastomataceae	Shrub	Wf	Fruit	Eat directly	–	8–12	8	1.5	2.0	1.50	0.5	6.5	3	3.5	LC	HRC1313
*Melastoma sanguineum* Sims	毛棯	–	Melastomataceae	Shrub	Wf	Fruit	Eat directly	–	8–12	42	2.0	2.0	1.50	0.5	6.5	3	24.6	LC	HRC1413
*Melicope pteleifolia* (Champion ex Bentham) T. G. Hartley	三桠苦	San cha ku	Ulmaceae	Tree	Ts	Leaf	Water-boiled	–	1–12	36	3.0	2.0	1.50	1	6.5	5	105.3	LC	HRC1416
*Melocalamus arrectus* T.P.Yi	澜沧梨藤竹	–	Poaceae	Bamboo	Wv	Tender stem	Stew soup	Stew with duck	12, 1–3	36	3.0	2.0	1.00	1	9	3	58.3	LC	HRC1253
*Mentha canadensis* Linnaeus	薄荷	Ge pa hen miao	Lamiaceae	Herb	Wv; Sp	Tender branches and leaves	Water-boiled、Stir-fry	Made into seasoning with chili, salt, and oil	1–12	108	1.5	4.0	1.50	0.75	10	4	255.2	LC	HRC1344
*Mentha spicata* L.*	留兰香	Gou rou xiang	Lamiaceae	Herb	Wv; Sp	Tender branches and leaves	Water-boiled、Stir-fry	Important seasonings for eating dry pot dog meat and lamb	1–12	55	1.5	4.0	1.50	1	10	3	129.9	LC	HRC1418
*Momordica cochinchinensis* (Lour.) Spreng	木鳖子	–	Piperaceae	Liana	Fd; Sp; Wv	Fruit; Young shoot	Friut: pound; Young shoot: water-boiled, stir-fry	After crushing, it is mixed with glutinous rice to make yellow Ciba	8–12, 2–4	85	3.0	4.0	1.50	1.5	7.5	5	753.1	LC	HRC1298
*Momordica subangulata* Blume	凹萼木鳖	–	Piperaceae	Liana	Wv	Fruit	Stir-fry	–	8–12	32	1.5	2.0	1.50	1	7.5	4	43.2	LC	HRC1419
*Murdannia bracteata (C,B. Clarke) J. K. Morton ex Hong*	大苞水竹叶	Shi zi cao	Commelinaceae	Herb	Wv; Ts	Whole plant	Water-boiled	Boil with lean meat	1–12	38	2.5	3.0	1.50	1.5	7.5	4	192.4	LC	HRC1268
*Musa balbisiana* Colla	野蕉	Ge gun nong	Musaceae	Herb	Wv	Pseudostem	Stir-fry	Stir-fry with meat	1–12	13	2.5	1.0	1.00	1.5	6.5	3	9.5	LC	HRC1310
*Nanhaia speciosa* (Champ. ex Benth.) J. Compton & Schrire	南海藤	Niu da li	Fabaceae	Liana	Lb	Root	Brewing	–	1–12	73	1.5	3.0	1.50	1	6.5	5	160.1	LC	HRC1420
*Nekemias grossedentata* (Hand.-Mazz.) J. Wen & Z. L. Nie	大齿牛果藤	–	Vitaceae	Liana	Ts	Branches and leaves; Stem	Water-boiled	–	7–12, 1	44	3.5	4.0	1.50	1	10	5	404.3	LC	HRC1269
*Nephrolepis cordifolia* (L.) C.Presl	肾蕨	–	Polypodiaceae	Herb	Wf	Rhizome	Eat directly	–	1–12	43	3.5	1.0	1.50	0.5	5.5	4	24.8	LC	HRC1421
*Ocimum basilicum* L. *	罗勒	Yue nan bo he	Lamiaceae	Herb	Sp	Whole plant	Eat directly	–	2–11	70	2.0	4.0	2.00	0.5	10	4	336.0	LC	HRC1249
*Oenanthe javanica* (Blume) DC	水芹	Shui qin cai	Apiaceae	Herb	Wv	Tender branches and leaves	Cold toss; Stir-fry	–	12,1–3	63	3.0	3.0	2.00	1.5	7.5	3	382.7	LC	HRC1303
*Oroxylum indicum* (L.) Kurz	木蝴蝶	–	Bignoniaceae	Tree	Wv	Seed	Stew soup	–	10–12, 1	6	2.0	2.0	1.00	0.75	7.5	5	6.8	LC	HRC1299
*Osbeckia chinensis* L	金锦香	–	Melastomataceae	Shrub	Ts	Whole plant	Water-boiled	–	1–12	16	2.5	2.0	2.00	1	6.5	5	52.0	LC	HRC1246
*Osyris lanceolata* Hochst. & Steud	沙针	–	Santalaceae	Shrub	Wf	Fruit	Eat directly	–	9–10	8	2.0	1.0	1.50	0.5	6.5	3	2.3	LC	HRC1422
*Paederia foetida* L	鸡屎藤	Ge hou tun ma	Rubiaceae	Shrub	Sn; Wv	Leaf	Water-boiled; Pound	Boil red sugar to make sugar water	3–5	80	3.0	2.0	1.50	1.5	7.5	4	324.0	LC	HRC1292
*Patrinia scabiosifolia* Link	败酱	Ge song pie	Caprifoliaceae	Herb	Wv	Tender leaf	Stew soup; Stir-fry	–	2–4	16	1.5	2.0	2.00	1	6.5	4	25.0	LC	HRC1423
*Pentaphragma spicatum* Merr	直序五膜草	Fei cui cai	Pentaphragmataceae	Herb	Wv	Leaf	Stir-fry、Stew soup	–	1–12	30	2.0	3.0	2.00	1	7.5	3	81.0	LC	HRC1265
*Perilla frutescens* var. *purpurascens* (Hayata) H. W. Li	野生紫苏	–	Lamiaceae	Herb	Wv; Sp	Leaf	Stir-fry	Made into dipping sauce with salt and oil, used as a dipping sauce for raw fish	1–12	76	2.5	3.0	2.00	0.75	9	4	307.8	LC	HRC1311
*Peristrophe bivalvis* (L.) Merr	观音草	Hong lan cao	Acanthaceae	Herb	Fd	Leaf	Pound	Crush and soak, then dye glutinous rice	3–4	68	2.5	2.0	2.00	1.5	7.5	3	229.5	LC	HRC1278
*Peristrophe japonica* (Thunb.) Bremek	狗肝菜	–	Acanthaceae	Herb	Fd	Branches and leaves; Stem	Pound; Stir-fry; Soak in boiling water	Crush and soak, then dye glutinous rice	3–4	52	3.0	1.0	2.00	1.5	7.5	4	140.4	LC	HRC1276
*Peristrophe japonica* (Thunb.) Bremek	九头狮子草	–	Acanthaceae	Herb	Fd	Branches and leaves; Stem	Soak in boiling water	Dyeing glutinous rice (blue color)	3–4	15	3.0	2.0	1.00	1.5	10	5	67.5	LC	HRC1334
*Persicaria viscosa* (Buch.-Ham. ex D. Don) H. Gross ex Nakai *	香蓼	–	Polygonaceae	Herb	Sp	Whole plant	Eat directly	Dipping sauce for raw fish	1–12	65	2.0	3.0	2.00	0.5	10	4	182.0	LC	HRC1322
*Phyllanthus emblica* L	余甘子	Meng han	Phyllanthaceae	Tree	Wf	Fruit	Eat directly	–	7–10	115	3.0	3.0	1.50	0.5	7.5	4	232.9	LC	HRC1315
*Phyllanthus urinaria* L	叶下珠	Ye cha zhu	Phyllanthaceae	Herb	Wv	Branches and leaves; Stem	Stew soup	Stew soup with duck liver	2–5	11	3.0	1.0	1.50	0.75	7.5	5	13.9	LC	HRC1424
*Phyllostachys heteroclada* Oliv	水竹	Xiao sun zi	Poaceae	Bamboo	Wv	Tender stem	Stir-fry	Stir-fry with meat and chili	3–4	21	2.0	2.0	1.00	1	7.5	3	18.9	LC	HRC1304
*Piper sarmentosum* Roxb	假蒟	Jia lou	Piperaceae	Herb	Wv; Sp	Leaf	Stir-fry; stew、Fry	Wrap the pork filling with leaves and fry it	1–12	100	3.0	3.0	2.00	1	10	5	900.0	LC	HRC1243
*Plantago asiatica* L	车前	Pa dui ma	Plantaginaceae	Herb	Wv; Ts	Whole plant	Stir-fry; Water-boiled; Pound	Crush and mix with glutinous rice flour to make Ciba	5–11	93	3.5	3.0	2.00	1	7.5	5	1098.6	LC	HRC1349
*Pleioblastus amarus (*Keng) Keng f	苦竹	Xiao sun zi	Poaceae	Bamboo	Wv	Tender stem	Stir-fry	Stir-fry with meat and chili	4–5	33	2.5	2.0	1.00	1	7.5	3	37.1	LC	HRC1450
*Pleuropterus multiflorus* (Thunb.) Turcz. ex Nakai	何首乌	–	Polygonaceae	Herb	Wv	Tender branches and leaves	Stir-fry; Cold toss	Cold toss with chili	2–3	11	3.5	2.0	1.50	1.5	6.5	5	56.3	LC	HRC1285
*Portulaca oleracea* L	马齿苋	Pa gou gei	Portulacaceae	Herb	Wv	Whole plant	Make filling; Cold toss	Making dumplings	5–9	93	2.5	4.0	2.00	1.5	7.5	5	1046.3	LC	HRC1251
*Praxelis clematidea* (Hieron. ex Kuntze) R.M.King & H.Rob. *	假臭草	–	Asteraceae	Herb	Wv	Tender leaf	Water-boiled; Stir-fry	–	3	6	2.5	1.0	0.75	1	6.5	3	2.2	LC	HRC1296
*Psidium guajava* L. *	番石榴	Ma dou wu	Myrtaceae	Shrub	Wf	Fruit	Eat directly	–	9–12, 1–3	106	3.0	2.0	1.50	0.5	7.5	3	107.3	LC	HRC1456
*Pteridium revolutum* (Blume) Nakai	毛轴蕨	Long zhua cai	Dennstaedtiaceae	Herb	Wv	Tender branches and leaves	Cold toss; Stir-fry; Stew soup	–	2–8	6	2.0	2.0	1.25	1.5	6.5	3	8.8	LC	HRC1456
*Pueraria montana* var. *thomsonii* (Benth.) M.R.Almeida	粉葛	Ge hou gan	Fabaceae	Liana	Wv	Root	Water-boiled; Pound	After crushing, soak in water, filter to extract starch and make into cakes	8–12, 1	90	3.0	1.0	1.50	1	7.5	4	121.5	LC	HRC1457
*Pyrus calleryana* Decne	豆梨	–	Rosaceae	Tree	Wf	Fruit	Eat directly; Brewing	–	8–11	21	2.5	2.0	1.50	1	6.5	4	41.0	LC	HRC1339
*Rhododendron simsii* Planch	杜鹃	Du jing	Ericaceae	Shrub	Sn	Flower	Cold toss	Eat by dipping in white sugar	4–5	9	3.0	1.0	0.75	1.5	9	3	8.2	LC	HRC1341
*Rhodomyrtus tomentosa* (Aiton) Hassk	桃金娘	Ma leng	Myrtaceae	Shrub	Wf	Fruit	Eat directly	–	6–7	131	3.5	2.0	1.50	0.5	6.5	4	178.8	LC	HRC1306
*Robinia pseudoacacia* L. *	刺槐	Yang huai hua	Fabaceae	Tree	Wv	Flower	Steam	Make rice cakes with glutinous rice flour	4–6	13	1.5	2.0	0.75	1	10	4	11.7	LC	HRC1459
*Rorippa indica* (L.) Hiern	蔊菜	Tang ga cai	Brassicaceae	Herb	Wv	Tender branches and leaves	Cold toss; Stir-fry; Stew soup	Stir-fry with eggs	2–3	25	2.5	2.0	2.00	1.5	7.5	3	84.4	LC	HRC1284
*Rosa laevigata* Michx	金樱子	Ma leng du	Rosaceae	Shrub	Lb	Fruit	Brewing	–	7–11	95	3.0	2.0	1.50	1	6.5	5	277.9	LC	HRC1333
*Rubus alceifolius* Poir	粗叶悬钩子	–	Rosaceae	Shrub	Wf	Fruit	Eat directly	–	9–11	30	3.0	2.0	1.50	0.5	7.5	3	30.4	LC	HRC1461
*Rubus cochinchinensis* Tratt	蛇藨筋	–	Rosaceae	Shrub	Wf	Fruit	Eat directly	–	6–7	31	2.5	2.0	1.50	0.5	7.5	3	26.2	LC	HRC1462
*Rubus leucanthus* Hance	白花悬钩子	–	Rosaceae	Shrub	Wf	Fruit	Eat directly	–	8–10	12	2.0	2.0	1.50	0.5	7.5	3	8.1	LC	HRC1463
*Rubus pluribracteatus* L. T. Lu & Boufford	大乌泡	–	Rosaceae	Shrub	Wf	Fruit	Eat directly	–	6	21	1.5	2.0	1.50	0.5	7.5	3	10.6	LC	HRC1272
*Rubus rosifolius* Stokes	空心藨	–	Rosaceae	Shrub	Wf	Fruit	Eat directly	–	7–9	82	3.5	2.0	1.50	0.5	9	3	116.2	LC	HRC1336
*Sageretia thea* (Osbeck) M.C.Johnst	雀梅藤	–	Rhamnaceae	Liana	Wf	Fruit	Eat directly	–	3–5	30	2.5	1.0	1.50	0.5	7.5	3	12.7	LC	HRC1330
*Salix babylonica* L. *	垂柳	Liu ye	Salicaceae	Tree	Wv; Ts	Young shoot	Cold toss; Stir-fry after drying; Soak in boiling water	–	2–3	15	2.0	1.0	1.25	1.5	7.5	4	16.9	LC	HRC1464
*Sarcandra glabra* (Thunb.) Nakai	草珊瑚	Ge sa ha ba bu	Chloranthaceae	Shrub	Ts	Whole plant	Water-boiled	–	1–12	116	3.5	3.0	2.00	1	7.5	5	1065.8	LC	HRC1348
*Saurauia tristyla* DC	水东哥	Ge ma mou	Actinidiaceae	Shrub or Dungarunga	Wf	Fruit	Eat directly	–	6–8	25	2.5	2.0	1.50	0.5	7.5	3	21.1	LC	HRC1465
*Scleromitrion diffusum* (Willd.) R.J.Wang	白花蛇舌草	–	Rubiaceae	Herb	Wv; Ts	Whole plant	Water-boiled	Cooking congee	3–7	62	3.0	2.0	2.00	1	6.5	5	241.8	LC	HRC1466
*Senegalia pennata* (L.) Maslin	羽叶金合欢	Chou cai	Fabaceae	Liana	Wv	Branches and leaves; Stem	Stir-fry; Stew soup	Stir-fry with eggs	3–4	16	2.5	1.0	2.00	1	10	3	24.0	LC	HRC1467
*Senna occidentalis* (L.) Link *	望江南	Ge du gua mei	Fabaceae	Shrub	Wv	Tender leaf	Stir-fry; Stew soup	Stew soup with chicken	3–4	58	3.0	2.0	0.75	1	10	4	104.4	LC	HRC1468
*Senna tora* (L.) Roxb. *	决明	Ge shu du dong	Fabaceae	Herb	Wv	Tender branches and leaves	Cold toss; SeedWater-boiled	Cook in hot pot	3–4,8–11	15	3.0	2.0	1.00	1.5	10	5	67.5	LC	HRC1335
*Solanum americanum* Miller	少花龙葵	Bai hua cai	Solanaceae	Herb	Wv; Wf	Tender branches and leaves	Water-boiled; Stir-fry; Cold toss	Stir-fry with meat	1–12	68	3.5	3.5	1.50	1	9	5	562.3	LC	HRC1472
*Striga asiatica* (L.) Kuntze	独脚金	Ge gan bu pie	Orobanchaceae	Herb	Ts	Whole plant	Soak in boiling water	–	5–8	25	1.5	2.0	2.00	1	6.5	5	48.8	LC	HRC1340
*Syzygium cumini* (L.) Skeels	乌墨	–	Myrtaceae	Tree	Ts	Flower	Soak in boiling water	–	2–3	16	2.5	2.0	0.75	1	7.5	5	22.5	LC	HRC1473
*Tadehagi triquetrum* (L.) H.Ohashi	葫芦茶	Ge sha hou bu	Fabaceae	Shrub	Ts	Whole plant	Water-boiled	–	1–12	42	3.0	2.0	2.00	1	7.5	5	189.0	LC	HRC1474
*Talipariti tiliaceum* (L.) Fryxell	黄槿	–	Malvaceae	Shrub or Dungarunga	Wv	Flower	Stew soup	Make soup with eggs	4–8	13	2.5	1.0	0.75	1	9	4	8.8	LC	HRC1288
*Taraxacum mongolicum* Hand.-Mazz	蒲公英	–	Asteraceae	Herb	Wv; Ts	Whole plant	Cold toss; Stir-fry; Water-boiled	–	2–8	32	1.5	2.0	2.00	1.5	9	5	129.6	LC	HRC1326
*Taxillus chinensis* (DC.) Danser	广寄生	–	Loranthaceae	Shrub	Ts	Branches and leaves; Stem	Water-boiled	–	1–12	53	3.0	3.0	2.00	1	6.5	5	310.1	LC	HRC1280
*Toona sinensis* (A.Juss.) M.Roem	香椿	Ge mei xia song	Meliaceae	Tree	Wv	Whole plant	Stir-fry; Cold toss; Fry	Stir-fry with eggs	3–4	43	3.0	1.0	1.50	1.5	10	3	87.1	LC	HRC1321
*Ulmus pumila* L	榆树	Yu qian	Ulmaceae	Tree	Wv	Fruit	Cold toss; Steam	–	3–6	6	1.5	1.0	1.50	1.5	7.5	3	4.6	LC	HRC1471
*Vernonia amygdalina* Delile *	扁桃斑鸠菊	Nan fei ye	Asteraceae	Shrub or Dungarunga	Ts	Leaf	Water-boiled	–	1–12	18	2.0	3.0	2.00	1	9	5	97.2	LC	HRC1347
*Viola philippica* Cav	紫花地丁	Ge deng ba kuo	Violaceae	Herb	Wv	Tender leaf	Cold toss	–	3–4	39	2.5	1.0	2.00	1.5	7.5	5	109.69	LC	HRC1469
*Viscum ovalifolium* Wall. et DC	瘤果槲寄生	–	Loranthaceae	Shrub	Ts	Branches and leaves; Stem	Water-boiled	–	1–12	5	2.0	2.0	2.00	1	6.5	5	13.00	LC	HRC1470
*Vitis balansana* Planchon	小果葡萄	Ge ma yi dong	Vitaceae	Liana	Wf	Fruit	Eat directly	–	9–10	39	2.5	1.0	2.00	1.5	7.5	5	109.69	LC	HRC1323
*Zingiber guangxiense* D. Fang	桂姜	Ye sheng jiang	Zingiberaceae	Herb	Wv; Sp	Pseudostem	Stir-fry; Boil in water; Stew soup	Stew fish with peeled stems; Stir-fry or Water-boil chunks of root and other meat together	3–4	19	1.5	2.0	1.50	0.75	10	4	25.65	LC	HRC1282
*Zingiber striolatum* Diels	阳荷	–	Zingiberaceae	Herb	Wv	Flower	Stir-fry	Stir-fry with meat	1–12	31	1.5	1.0	0.75	1	10	5	17.44	LC	HRC1308

The WEPs used by Zhuang people include wild vegetables (Wv), wild fruits (Wf), nuts (Nu), spices (Sp), substitutes (Ts), liquor brewing (Lb), oils and fats (Of), snack (Sn)and food dyeing (Fd)

*Refers to alien species and spontaneous; *LC* least concern, *EN* endangered, *Vu* vulnerable

**Fig. 2:**
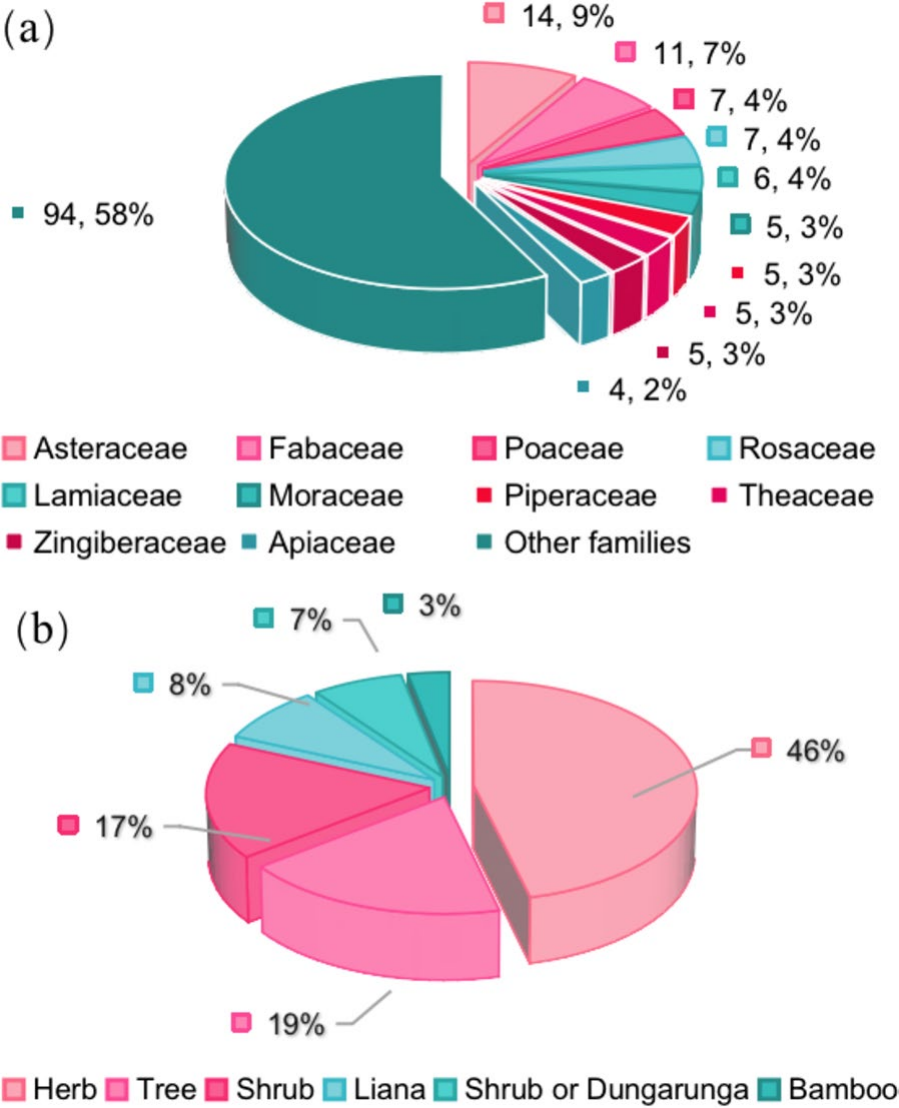
**a** Family distribution of WEPs species of angiosperm category; **b** Habitats of WEPs used by Zhuang people

These species belong to 67 botanical families, of which the largest are Asteraceae (14 species), Fabaceae (11), Poaceae (7) and Rosaceae (7) (Fig. 2a). There are 35 families with only one species. The habitats of these WEPs are mostly herb (74) followed by trees (30), shrubs (27), Liana (16), Shrub or Dungarunga (11) and Bamboo (5) (Fig. 2b). Local consumption of rare and endangered plants is not common, with three species classified as “Endangered” and three more categorized as “Vulnerable”, while the remaining species are considered “Least Concern”. Furthermore, it is worth noting that 17 of these wild edible plant species are non-native, as indicated by asterisks in Table 2. Out of these, seven species have been identified as invasive alien species.

**Fig. 3:**
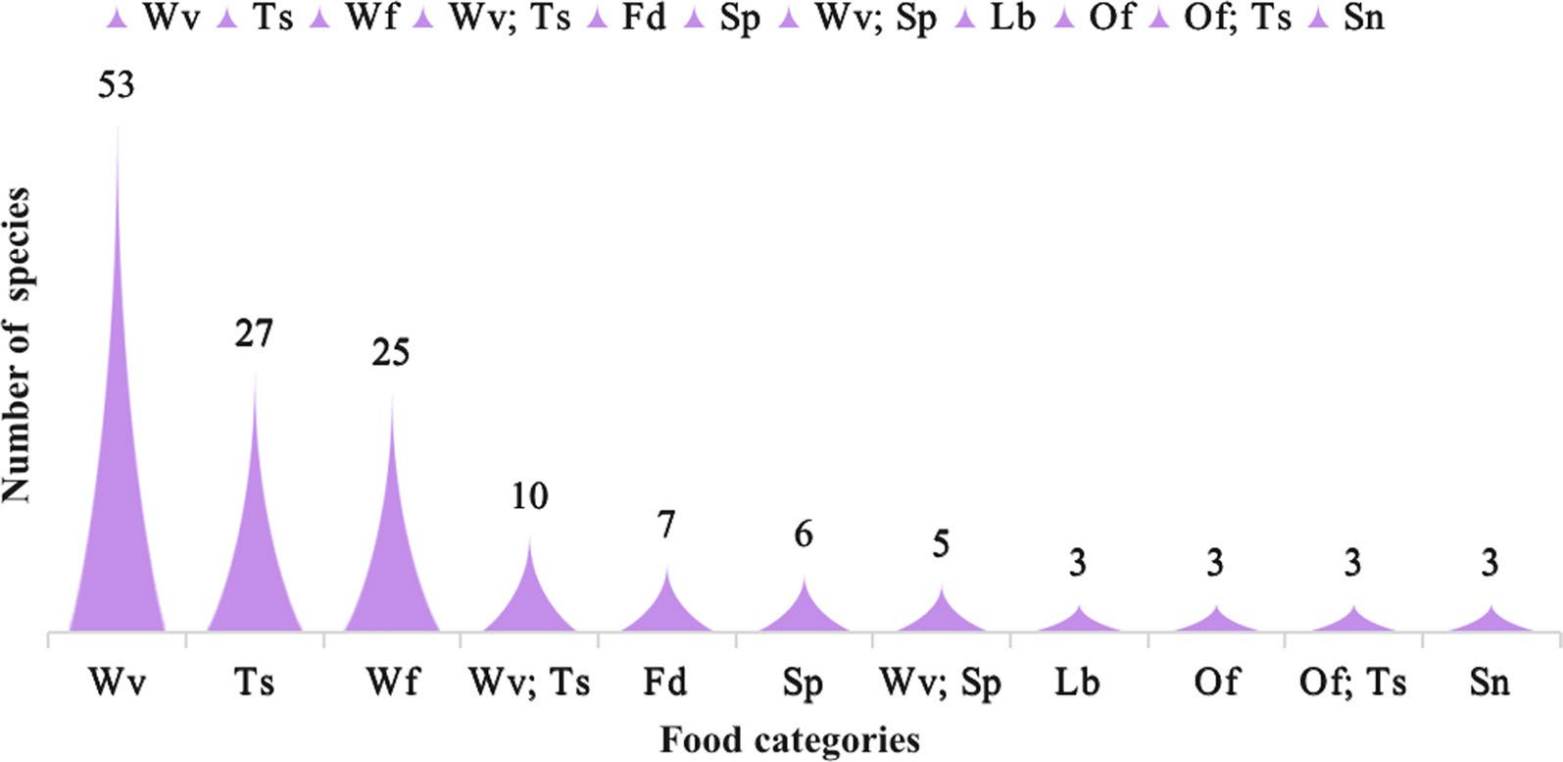
Main food categories of WEPs used by Zhuang people (Abbreviations in this figure are the same as those in Table 2)

**Fig. 4:**
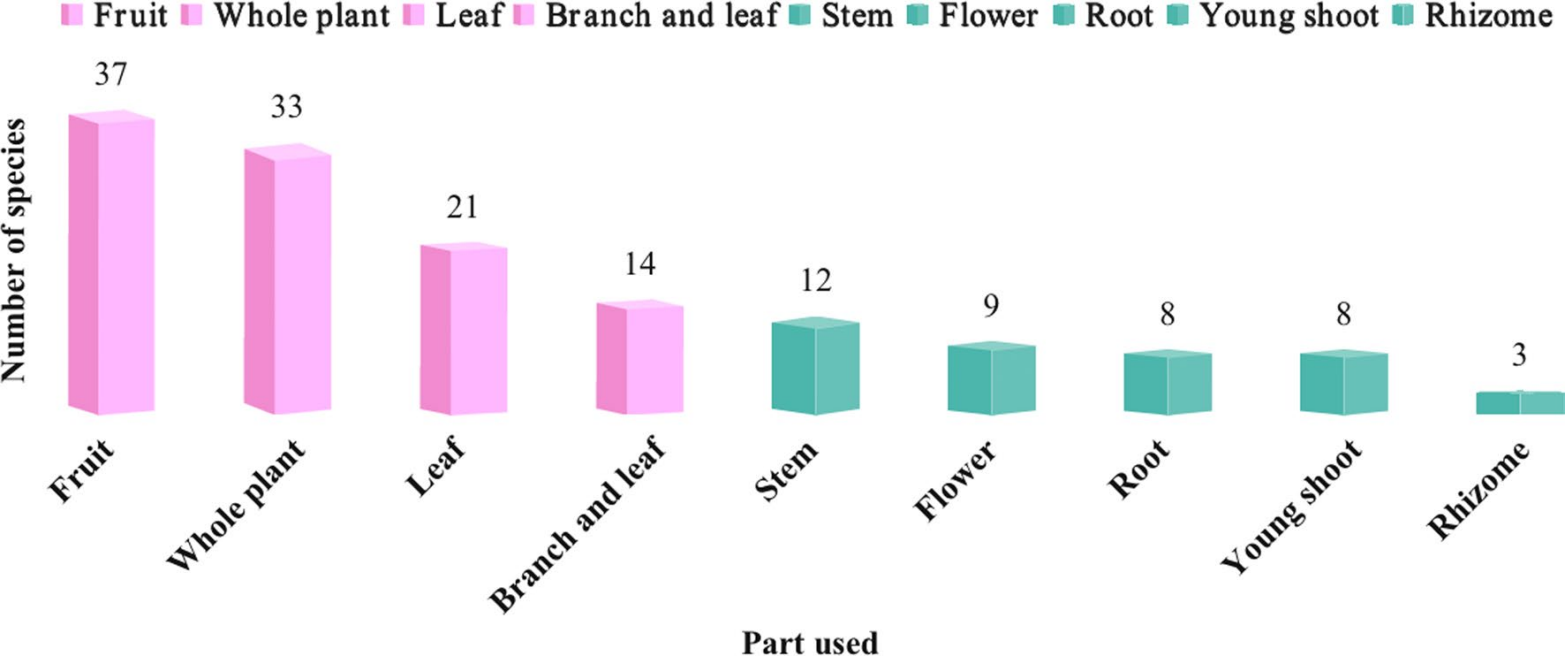
Main edible parts of WEPs used by Zhuang people

**Fig. 5:**
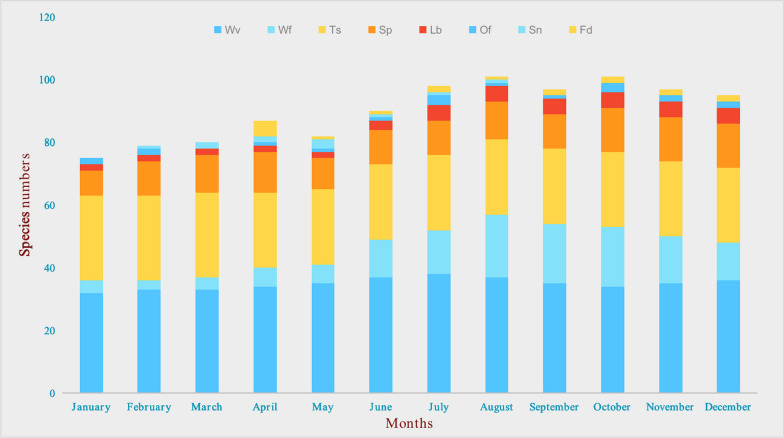
Months of collecting WEPs

Wild vegetables and tea substitutes are two main categories of WEPs (Fig. 3). All WEPs are typically consumed in the form of stir-fried dishes, boiled items, soups, served cold, and dressed in sauces. Plants have edible parts, such as stems, leaves, fruits, seeds, flowers, roots, and tubers. Among them, the most commonly consumed parts are fruits (37, 22.7%), followed by whole plants (33, 20.2%) and leaves (21, 12.9%) (Fig. 4). The availability of wild edible plants remains high throughout the year, with peak seasons in August (101) and October (101), followed closely by July (98) and November (96). The lowest abundance is observed in January (75), with a difference of 26 plant species compared to the highest abundance in August and October. Therefore, the period from July to November is considered the most suitable for consumption of wild edible plants in Fangchenggang. During this time, the market also offers the widest variety of plant species for sale (Fig. 5).

### Wild vegetables

**Fig. 6:**
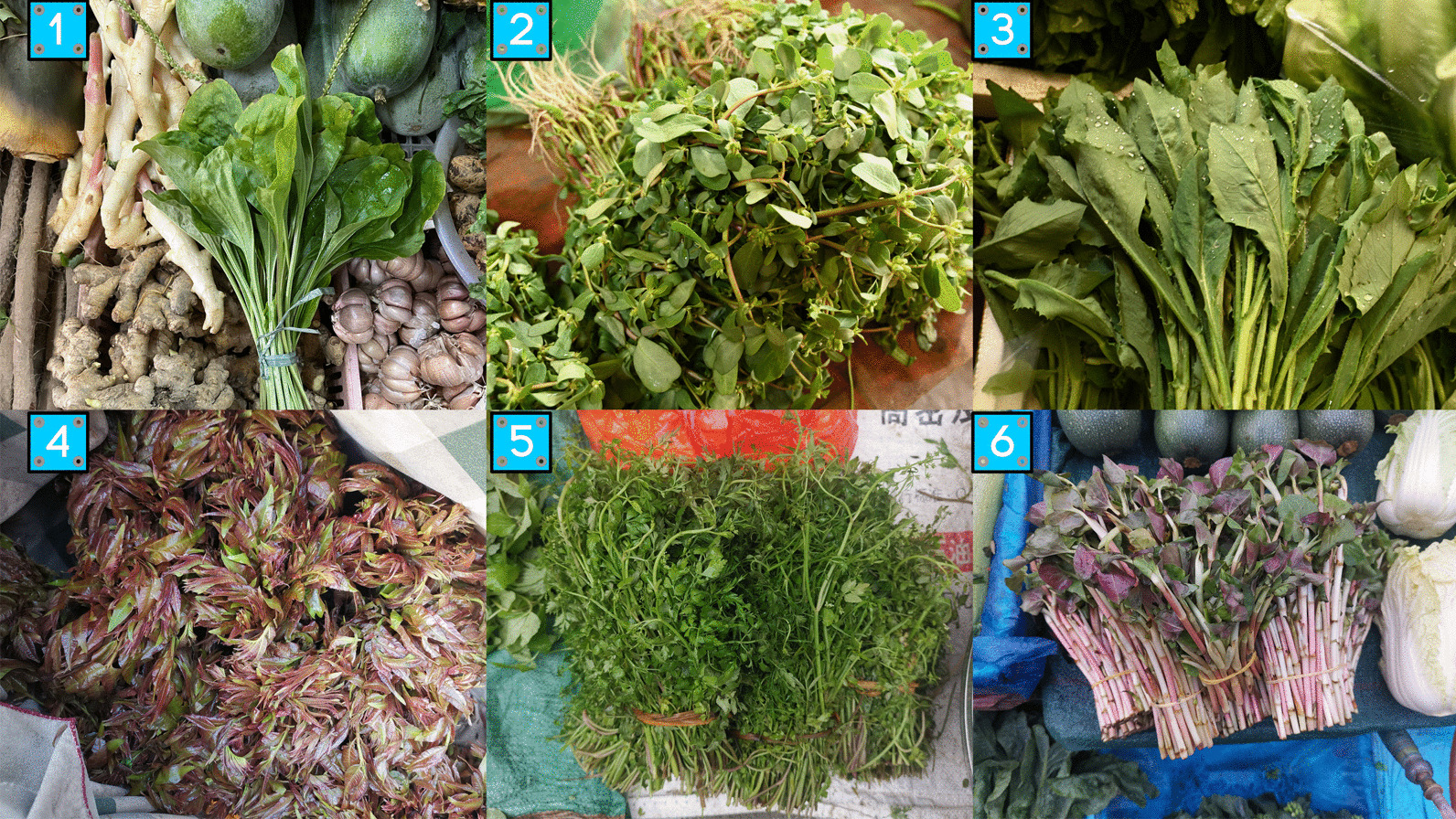
Some wild vegetables in the study area [(1) *Plantago asiatica*; (2) *Portulaca oleracea*; (3) *Emilia sonchifolia*; (4) *Toona sinensis*; (5) *Oenanthe javanica*; 6.*Houttuynia cordata*]

Wild vegetables were the most extensively utilized food category, comprising 53 edible species. Additionally, there were ten plants that served as both vegetables and tea substitutes, five species that functioned as both vegetables and spices, one plant that served as both a vegetable and a nut, and one plant that served as both a vegetable and a fruit, making a total of 69 species belonging to 40 families. The primary edible parts of wild vegetables are the whole plant (34), tender branches and leaves (21). They are commonly consumed in diverse forms such as salads (after treatment), cooking in hot water, or stir-frying. Additionally, they are used as ingredients in soups or when stewed with pork or chicken. It is not common to consume wild raw vegetables without pre-treatment (Fig. 6).

Wild vegetables are typically priced slightly higher than conventional vegetables, such as *Pentaphragma spicatum*, which is an endemic species of China and belongs to the family Pentaphragmataceae. This plant species is found exclusively in southern Guangxi, including Shiwan Mountains, Dongxing, Fangcheng, Daxin, Xinyi in Guangdong, and Baisha and Baoting on Hainan Island [34]. It thrives in the dense forests of tropical valleys. Prior to this research study, there were no reports on the edibility of this plant. However, in Shangsi, local residents refer to this plant as a “jade vegetable” due to its jade-like edible leaves. The leaves can be picked and stored for up to a month without deterioration and can be stir-fried or used in soups. At the tourist restaurant of Shanwan Mountains in Shangsi County, there is a special dish called “Stir-Fried Jade Vegetable” served to visitors. The best taste of *P. spicatum*, nurtured by Shiwan Mountains, is achieved when it is stir-fried. Its flesh-like leaves are crisp and fragrant. The villagers who collected *P. spicatum* shared with us that consuming this plant can serve as a natural source of iron. Additionally, they mentioned that boiling the whole plant in water and using the resulting infusion externally may be effective in treating rheumatism, bruises, promoting blood circulation, and alleviating blood stasis [35]. The local residents near Shiwan Mountains are familiar with this plant and know precisely where it grows. However, due to the high environmental requirements of *P. spicatum*, villagers have been unsuccessful in transplanting it to their courtyards despite various attempts.

**Fig. 7:**
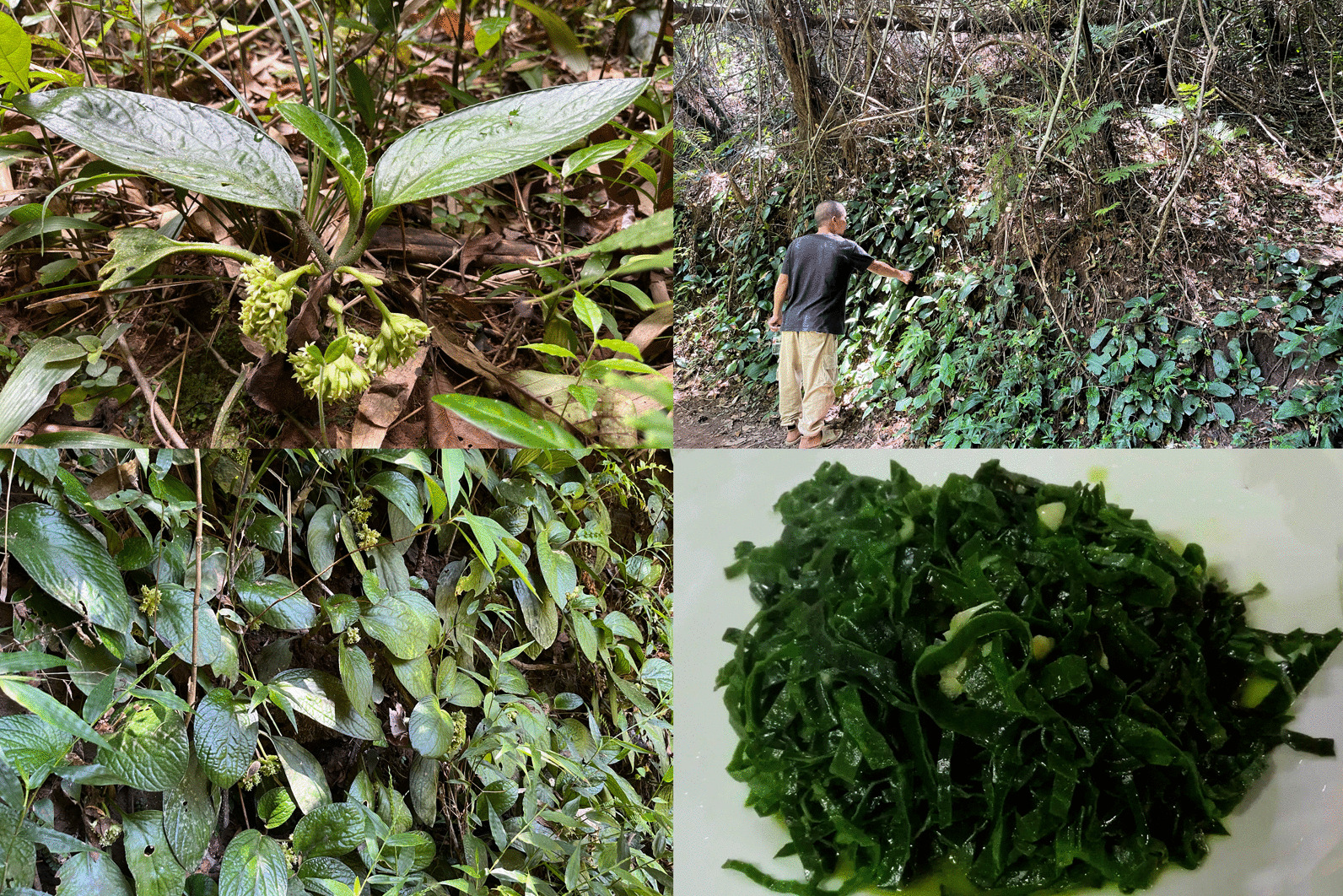
The habitat of *Pentaphragma spicatum* and dishes made from it

Consequently, the limited utilization of *P. spicatum* as a source of domesticated species and valuable genetic resources for developing new crops through hybrid screening naturally [36] results in higher prices (Fig. 7).

### Tea substitutes

**Fig. 8:**
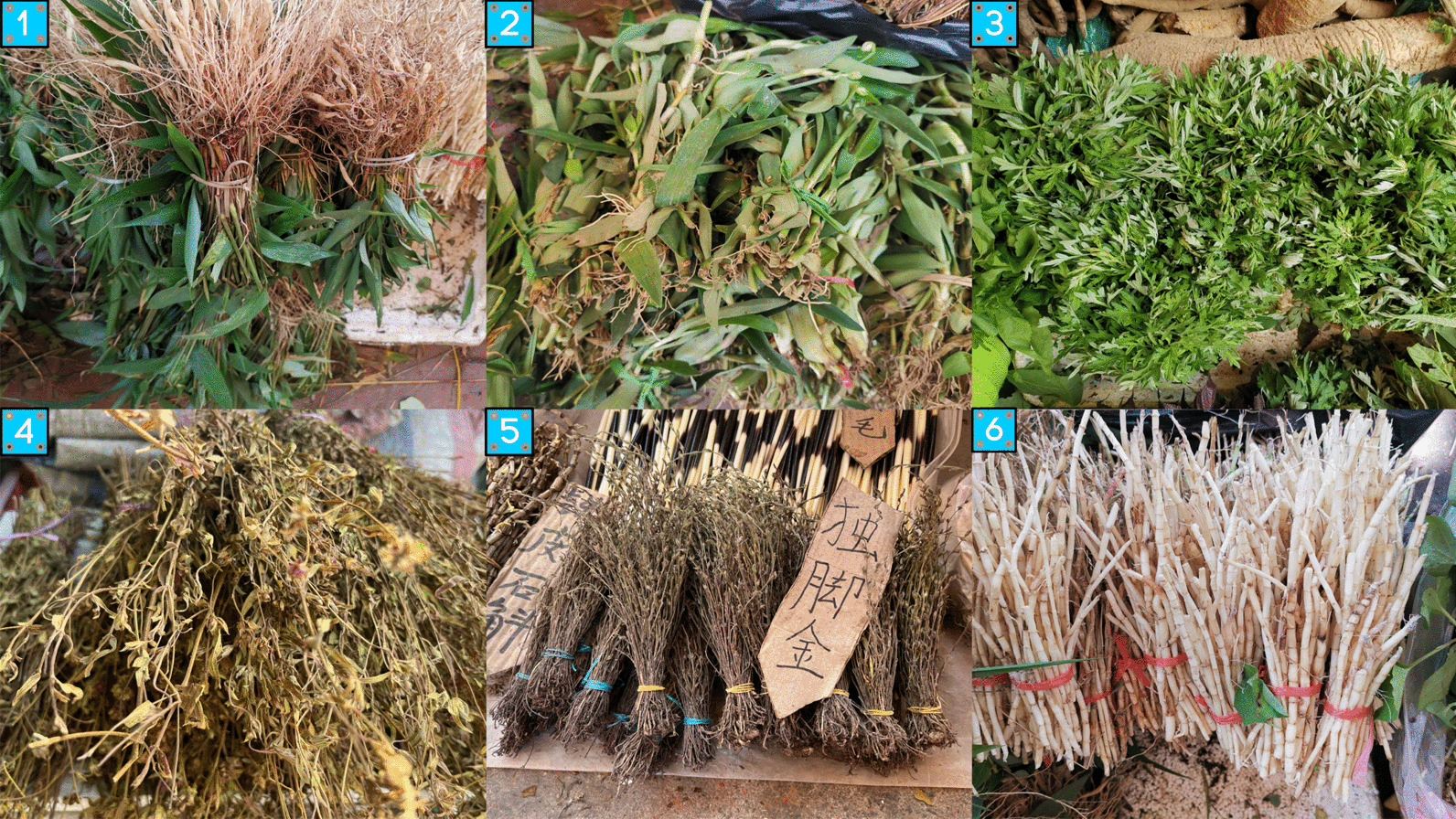
Some Liáng chá plants in the study area [(1) *Lophatherum gracile*; (2) *Murdannia bracteata*; (3) *Artemisia indica*; (4) *Osbeckia chinensis*; (5) *Striga asiatica*; (6) *Imperata cylindrica*]

Tea substitutes are the second largest food category of wild edible plants (WEPs) used by the Zhuang people, consisting of 42 species. Among them, 10 species serve as both wild vegetables and tea substitutes, while 3 species serve as both oils and fats and tea substitutes. The whole plants (21) of wild species are the most commonly used part, followed by the leaves (19). The usual method of preparation involves boiling in water or steeping in hot water. The tea substitute plants in the Fangchenggang region can be classified into two main types based on their different uses: Liáng chá and flavor-enhancing tea substitutes. There are 27 plant species such as *Sarcandra glabra*, *Centella asiatica*, and *Gynostemma pentaphyllum*, which are used by local residents as Liáng chá to cope with hot weather. Another 15 species like *Camellia petelotii*, *Camellia euphlebia*, and *Helixanthera parasitica* are chosen by Zhuang people living in remote areas when tea is not readily available, in order to enhance the flavor by steeping these plants (Fig. 8).

In eight different markets, a total of 24 vendors offer both fresh and dried Liáng chá plants. These vendors are distributed as follows: 6 in Shangsi County, 9 in Fangcheng District, 5 in Gangkou District, and 4 in Dongxing City. Customers have the flexibility to purchase specific plant species or opt for pre-mixed blends of Liáng chá. *Sarcandra glabra*, *Centella asiatica*, and *Gynostemma pentaphyllum* are the most commonly available plants used for making Liáng chá. Similar findings can also be found in the "Ethnobotanical study on herbal tea drinks in Guangxi, China” [37].

Some vendors prepare *Centella asiatica* with added sugar to create a refreshing tea beverage, which is sold at a price of 10 yuan per cup. Moreover, they also boil *Abrus pulchellus*, *Grona styracifolia*, *Siraitia grosvenorii* (cultivated, not listed in the inventory), *Lonicera japonica* (cultivated, not listed in the inventory), and *Hedyotis effusa* mixed with sugar to create a tea drink known as “Xia huo cha”, which helps to cool down the body. The price of this beverage is the same as that of *Centella asiatica*.

**Fig. 9:**
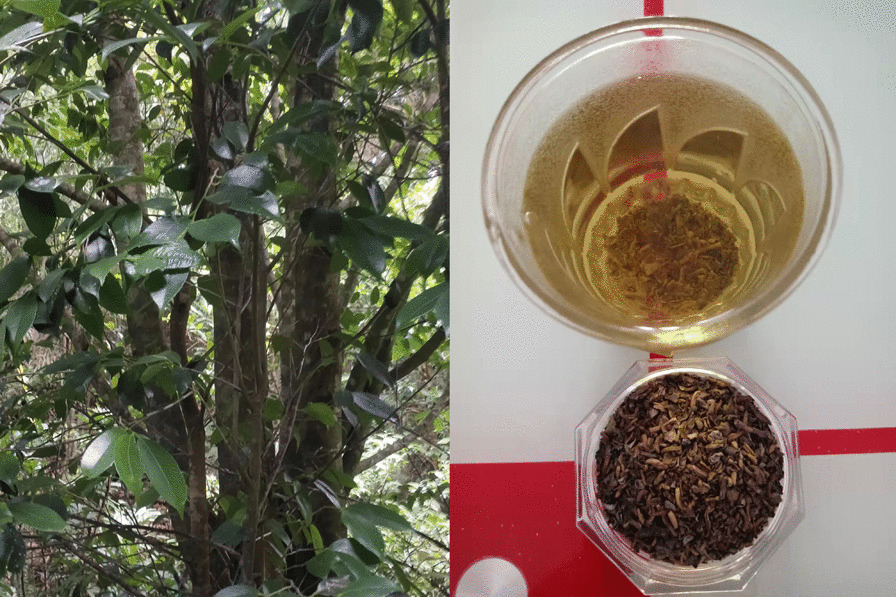
*Ilex confertiflora* and Qingming tea

*Ilex confertiflora*, known as “Qingming tea” among the Zhuang people in Shangsi County, is a highly regarded tea substitute plant traditionally harvested during the Qingming Festival. Interviewees have praised its exceptional taste, with a TSAI score of 9.0. However, in recent times, many younger individuals have migrated for employment opportunities, making them less inclined to invest their time in gathering and processing these plants in mountainous areas. Consequently, this traditional practice is primarily upheld by the older generations. Considering the plants in the *Ilex* genus of the Aquifoliaceae family often possess anti-inflammatory activities and other medicinal properties [38, 39], we interviewed 28 households and 71 individuals, with 56% of respondents stating that it has the effect of clearing heat and detoxification, 24% claiming it can lower blood pressure, and 20% being unsure about its specific effects. Further studies on its chemistry, biological activity, and toxicity are needed for potentially developing new tea substitute (Fig. 9).

### Wild fruits

**Fig. 10:**
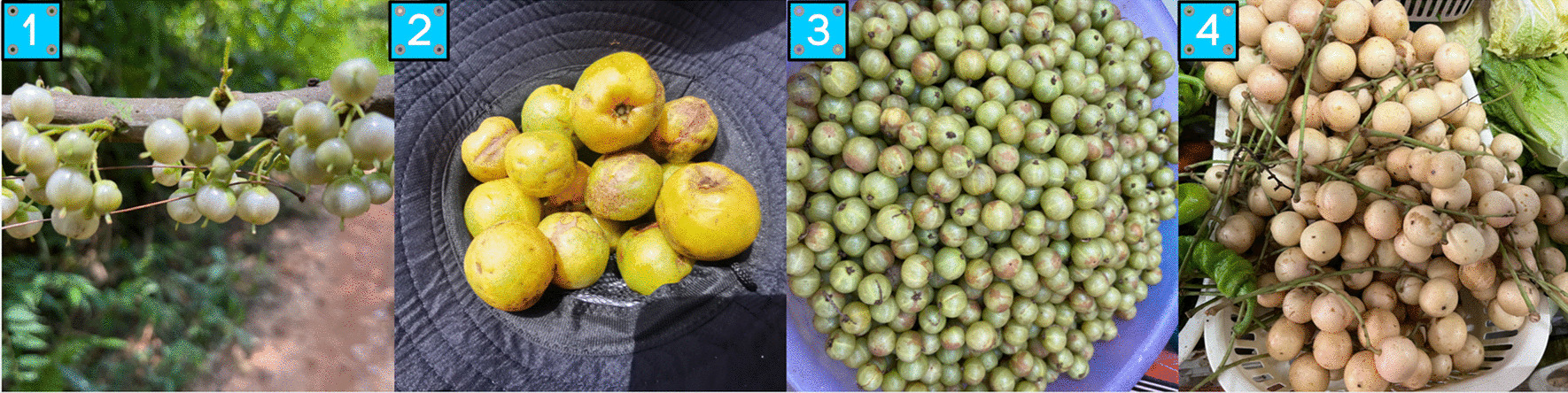
Some wild fruits in the study area [(1) *Saurauia tristyla*; (2) *Garcinia oblongifolia*; (3) *Phyllanthus emblica*; (4) *Baccaurea ramiflora*]

A total of 30 plant species are consumed as fruits. Among them, 3 species serve as both vegetables and fruits, while 1 species is considered both a nut and a fruit. The majority of the fruits we commonly consume in our daily lives are cultivated within the Rosaceae family [40]. Similarly, among the wild fruits surveyed, the Rosaceae family has the highest diversity of plant species, with many of them being shrubs that are easily gathered, such as *Rubus leucanthus*, *Rubus cochinchinensis*, and *Rubus pluribracteatus*. Many studies have revealed that wild fruits possess a higher nutritional value compared to cultivated fruits [41, 42]. Wild fruits are especially popular among children as they serve as a source of essential vitamins and minerals, particularly when cultivated fruits are not readily available. Additionally, *Saurauia tristyla* and *Phyllanthus emblica* are also commonly sold on the market (Fig. 10).

In addition to being consumed as fresh fruit, the wild fruits abundant in the area are traditionally fermented along with rice by the Zhuang people, such as *Rhodomyrtus tomentosa*, *Rosa laevigata*, *Vitis balansana*, and *Ficus hirta*, to produce a traditional alcoholic beverage known as “guo jiu”. This type of liquor typically has a lower alcohol content, usually around ten degrees. Local inhabitants believe that these beverages can promote blood circulation, stimulate metabolism, and have a beneficial effect on the body. Scientific research has also indicated that such as mature fresh fruits of *Rosa laevigata* contain high levels of vitamin C, reaching up to 1187.38 mg/100 g. Additionally, they have a total sugar content of 25.76%, with a reducing sugar content of 24.38% [43]. The fruit pulp contains 19 types of amino acids, including 8 essential amino acids for the human body. Transforming these fruits into alcoholic beverages can confer health benefits due to their nutritional composition [44].

### Spices

**Fig. 11:**
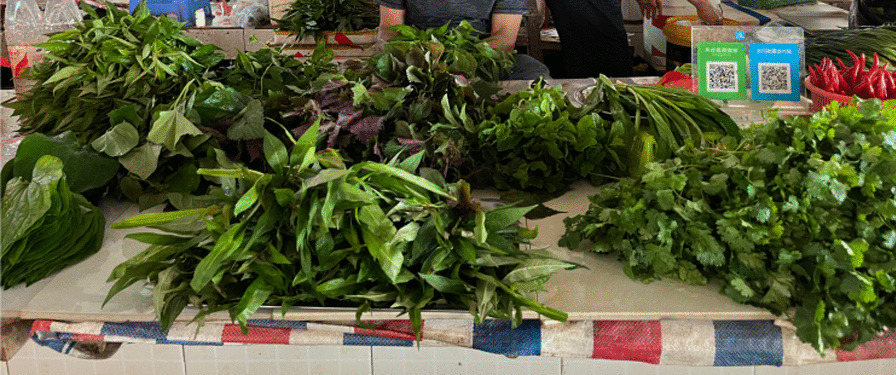
Stalls selling only spice plants

While the variety of wild spice plants is not extensive, their frequency of use is remarkably high, with a total of 15 species. Among them, 6 plants serve as both wild vegetables (Wv) and spices (Sp), and 1 plant also functions as wild fruit (Wf). Most of these spice plants are well-suited to complement local cuisine's unique flavors, offering elements of acidity, umami, and taste enhancement (Fig. 11).

One particularly noteworthy combination of spices is used to make a dipping sauce for sashimi (thin slices of raw fish). It typically includes *Piper sarmentosum*, *Persicaria viscosa*, *Perilla frutescens* var. *purpurascens*, *Houttuynia cordata*, and *Ocimum basilicum*. This combination is commonly found in the market (there are 18 vendors in total), where vendors often sell these plants bundled together. It is rare to find them sold separately, and some stalls specialize exclusively in these spice plants, highlighting their importance in the local cuisine. In more remote villages located far from the city, people also prepare this delicacy during festivals and special occasions. Although the condiments used may not be as diverse as those in urban areas, *Piper sarmentosum*, *Persicaria viscosa*, *Houttuynia cordata*, and *Ocimum basilicum* are essential ingredients that can be easily collected.

### Quantitative evaluation of Zhuang edible wild plants in Fangchenggang

The comparison results of the cultural food significance index (CFSI) of Zhuang edible wild plants in Fangchenggang are shown in Table 2. The edible wild plants in this area were clustered based on the CFSI, and those with broad application and high value, which played an important role in the local people's traditional diet, are highlighted. To classify the plants cited plants into four groups: species with very high significance (CFSI > 500), high significance (500 > CFSI ≥ 100), moderate significance (100 > CFSI ≥ 10), low significance (CFSI < 10).

In the very high significantcategory (CFSI > 500), there were a total of 15 plant species identified. Tea substitutes were the primary plants within this category, followed by vegetables. The notable plants in this category include *Camellia oleifera*, *Plantago asiatica*, *Sarcandra glabra*, *Portulaca oleracea*, *Centella asiatica*, *Piper sarmentosum*, and *Elephantopus scaber*. They are widely distributed in this area and are found almost everywhere.

*Camellia oleifera*, displaying the highest CFSI (The cultural food significance index) value, is extensively distributed in the Fangchenggang region. During periods of limited availability of edible oil, it has traditionally served as a source for oil extraction to meet daily dietary requirements. Contemporary scientific researches have revealed that *C. oleifera* oil possesses an unparalleled concentration of unsaturated fatty acids, ranging from 85 to 97%, thereby surpassing various other edible oils [45]. Its consumption has been linked to the effective prevention and treatment of cardiovascular and cerebrovascular ailments [46], leading to its recognition as the “oil of longevity”. Consequently, individuals persist in favoring *C. oleifera* oil due to its beneficial impact on health and its nutritional value.

A total of 53 plant species were classified in the high significant category (500 > CFSI ≥ 100). These species exhibit wide distribution in the area and offer a diverse range of fruits, vegetables, food dyes, snacks, and substitutes for tea to the local inhabitants. The relatively lower CFSI value of these plants is primarily attributed to factors such as the edible parts, taste, and the extent of domestication and cultivation carried out by the local residents. For instance, frequently encountered species like *Basella alba*, *Ocimum basilicum*, and *Curcuma longa* are predominantly cultivated, although not to the same extent as common staple vegetables. However, people also enjoy consuming them due to their high nutritional value [47].

Additionally, there are specific plants utilized for dyeing purposes, including *Liquidambar formosana*, *Curcuma longa*, *Peristrophe japonica*, and *Asystasia nemorum*. While these plants also hold significance in local wild vegetable consumption, they are predominantly associated with significant festivities and ceremonies rather than daily meals, such as the Dragon Boat Festival, Spring Festival, and weddings. Particularly on the third day of the third lunar month within the Zhuang ethnic group. Dyeing plants are indispensable components during these occasions [48]. The naturally occurring seasonal plants are utilized to dye glutinous rice into four colors: red, black, purple, and yellow. As glutinous rice is inherently white, only four types of dyeing plants are involved. Most Zhuang people used similar plant-based dyes (pigments), although slight variations may occur due to differences in the availability of plant resources in different regions. Additionally, the five-colored glutinous rice holds multiple significances, serving as both a festive delicacy and an offering to ancestors, symbolizing familial unity and harmony. The skill to make five-colored glutinous rice is possessed by every household, indicative of the widespread adoption and preservation of this culinary tradition [49]. Despite the time constraints faced by contemporary youth in preparing five-colored glutinous rice, they still purchase it from markets or restaurants. In comparison with other traditional cultural practices, the production and consumption of five-colored glutinous rice have been relatively well-maintained.

There were a total of 72 plant species classified in the moderate significant category (100 > CFSI ≥ 10). Herbaceous plants accounted for the highest number in this category. Moreover, several plants with medicinal properties were included, such as *Bidens pilosa*, and *Garcinia oblongifolia* and *Cibotium barometz*. In the low significant category (10 > CFSI), the number of plants was the lowest, with a total of 18 species. The plants in this category primarily consisted of species with special distribution areas, unappealing taste, or specific uses. An example of such a plant is *Praxelis clematidea*. In previous hard times, it was common to rely on this plant as a source of sustenance. However, with the availability of more options nowadays, it is frequently utilized as animal feed, particularly for pigs.

## Discussion

### The dietary habits and homology of medicine and food

The concept of “Homology of medicine and food” highlights that certain foods can fulfill basic nutritional needs but also possess medicinal properties similar to herbs, plants, or traditional remedies. This idea has its roots as far back as the Zhou Dynasty in China when a clear distinction between “food” and “medicine” was recognized. Over time, this gave rise to a specialized field known as “dietary therapy”, which focuses on the utilization of food as a form of medicine. In the Fangchenggang region, there exists a rich tradition of utilizing food for its therapeutic benefits [50].

### Liáng chá

Liáng chá has a long-standing tradition in Chinese culture and is known for its health-promoting effects. In this study, the Zhuang people frequently mention the health benefits of Liáng chá, especially its ability to alleviate heat from the body [51, 52], which is influenced by the environment. These findings are consistent with previous research that has explored the medicinal properties of Liáng chá [53–56]. The choice of ingredients for Liáng chá depends on an individual's constitution and the need for balancing yin and yang in the body. These ingredients can be categorized into “single herbs” and “compound herbs.” Single herb Liáng chá recipes are relatively simple, whereas compound Liáng chá recipes can be highly diverse, combining multiple herbs to create specific effects. Each Liáng chá recipe possesses its unique combination of medicinal properties and therapeutic effects, contributing to the distinctiveness of every family's formula [57]. Consequently, the knowledge and expertise in Liáng chá preparation have been passed down through generations, leading to the establishment of numerous time-honored Liáng chá shops in the local area, often kept within the same families for many years.

Our investigation found few adverse events associated with the most mentioned Liáng chá. However, it is crucial to be cautious as “natural” does not always mean safe. Studies have shown that excessive consumption of certain herbal teas can have negative effects, and some phytochemicals in herbal teas may pose health risks [58]. To ensure Liáng chá product safety, close monitoring of content and quality throughout the supply chain is vital, from collection and transportation to processing, production, and storage. Advanced technologies like two-dimensional chromatography fingerprinting, molecular identification, and chemical detection can help identify contaminants and adulterants in Liáng chá plant species.

### Dietary therapy

Several research findings have consistently demonstrated that wild vegetables generally offer higher nutritional and edible value compared to cultivated vegetables [58–61]. The cultural food significance index (CFSI) of Zhuang edible wild plants in Fangchenggang indicates that nearly all wild vegetables categorized under “very high significance” possess dual properties, serving both as medicinal herbs and edible food sources. For example, *Plantago asiatica* offers various health benefits, including antibacterial, anti-inflammatory, and anti-tumor properties. It also supports immune regulation and kidney regeneration [62]. The Zhuang people frequently prepare it in a stew with pig tripe to address jaundice and support good health. *Portulaca oleracea* can boost the human immune system and aid in the prevention and treatment of diseases such as heart disease, hypertension, and hyperlipidemia [63]. People prepare dish by blanching fresh and tender *P. oleracea* leaves in boiling water, cutting them into segments, and seasoning them with minced garlic, soy sauce, and sesame oil. Experimental studies have shown that *Centella asiatica* flavonoids have diverse physiological effects, including anticarcinogenic actions against tumors, antioxidative properties, antibacterial activity, wound healing acceleration, heat-clearing and detoxifying effects, as well as anti-inflammatory and bruise-treating capabilities [64]. Local residents not only stir-fry to preserve its freshness but also combine it with meat.

In addition, the method of soup preparation is a preferred way to benefit one's health. For example, the roots and stems of *Ficus hirta* are commonly used in soups with ingredients like chicken and pigeon, known for their potential to nourish the lungs, alleviate coughs, strengthen the spleen and stomach, promote beauty, aid in weight loss [65]. *Campanumoea javanica* soup is beneficial for invigorating the spleen and *Qi*, nourishing the lungs, and reducing coughs while promoting lactation [66]. *Nanhaia speciosa*, when boiled with pork ribs, is highly suitable for individuals with weak constitutions.

However, current research on the development and utilization of wild vegetables tends to emphasize their nutritional and medicinal value while not adequately addressing safety concerns related to their consumption [67, 68]. Influenced by environmental pollution, some wild vegetables may accumulate nitrites, heavy metals, and other contaminants during processing [69], while others may contain alkaloids that can be toxic if consumed in excess, leading to potential food poisoning if inadvertently consumed [70].

Therefore, it is crucial to enhance research efforts concerning the safety of consuming wild vegetables. Adhering to national food safety standards, rigorous analysis, testing, and safety assessments should be conducted on wild vegetables with high CFSI values. Additionally, scientific guidelines for the safe consumption of wild vegetables should be developed and widely disseminated to the public, increasing their awareness of safe foraging and consumption practices. Ensuring consumer confidence in the safety of wild vegetables will facilitate their widespread promotion and publicity without any apprehensions.

### Influence of border trade and exchange on the consumption of wild plants

Fangchenggang, with its subtropical monsoon climate and abundant rainfall, provides an ideal environment for the proliferation and growth of non-native plant species [71]. Additionally, strategically located as a border city between China and Vietnam, Fangchenggang benefits from its five first-class national ports [20], serving as a pivotal hub for Guangxi's participation in the “Belt and Road Initiative.” The increased trade activities in recent years have led to greater movement of people and goods, resulting in the introduction of numerous species into new regions, both intentionally and unintentionally. Based on this study, a total of 17 wild edible plant species in Fangchenggang belong to non-native species, and among them, 7 species have been listed as invasive alien species. Plants like *Basella alba*, *Bidens pilosa*, *Eryngium foetidum*, and *Amaranthus spinosus* have become highly popular wild vegetables in Fangchenggang. Moreover, some of these plants are now being cultivated. Therefore, border trade activities have facilitated the exchange and sale of wild plants from different regions, resulting in an increased supply and diversity of wild plants, leading to their more widespread consumption. Plants such as *Basella alba*, *Ocimum basilicum*, *Eryngium foetidum*, *Amaranthus tricolor*, and *Mentha spicata* all rank in the “high significance” category according to the cultural food significance index (CFSI) analysis. This highlights the significant roles these exotic species play in the local dietary habits.

From an ecological perspective, the consumption of exotic invasive species by people may help alleviate ecological pressure, reduce their population and spread. Furthermore, through the collection and consumption of these invasive species, people also develop effective management and conservation attitudes toward them [72]. In market, *Amaranthus spinosus* and *Basella alba* have become the cultivated vegetables. When people perceive these invasive species as valuable resources rather than merely harmful ones, they are more likely to actively participate in their management and control.

Also, the interaction between different ethnic groups has played a role in spreading and exchanging knowledge about locally consumed wild plants. Such as *Ocimum basilicum* and *Eryngium foetidum. E. foetidum* is a vital ingredient in a local Jing cuisine snack known as “qu tou dan”. Due to Dongxing's close proximity to Vietnam across the sea, the Zhuang people commonly refer to *O. basilicum* and *E. foetidum* as “Vietnamese mint” and “Vietnamese coriander”, respectively. However, our research and interviews indicate that the terms “Vietnamese mint” and "Vietnamese coriander" have only recently emerged, primarily driven by the development of tourism.

The situation differs from the challenges faced in conserving traditional medicine [73]. A significant portion of our interviewees falls within the 30–60 age group, with comparable knowledge levels regarding wild edible plants among both genders. The practice of consuming wild edible plants is deeply ingrained in the local culture. However, it is noteworthy that individuals under the age of 30 exhibit relatively limited familiarity with these plants, warranting attention.

### The ecological-cultural adaptation of wild edible plants

Food serves as a tangible representation of cultural heritage, and the development of the culture surrounding the consumption of wild edible plants in the Zhuang community of Fangchenggang is shaped by the longstanding interplay between geographical and cultural factors. This cultural phenomenon is a product of the reciprocal adaptation and mutual benefits between the local culture and the ecological environment.

### Freshness

Before 1950s, Fangchenggang was under the jurisdiction of Guangdong Province [74], thus inheriting the culinary traditions of Cantonese cuisine. The region's coastal location, along with its proximity to the Shiwan Mountains, has resulted in a wide availability of fresh wild plants throughout the year (Fig. 5), becoming a symbol of “freshness” highly valued by the local community. Almost all wild vegetables are processed using simple methods such as stir-frying and making soups (Table 2), with a focus on minimal processing to maintain the natural taste and texture of the fresh wild vegetables.

One notable aspect of the “freshness” (refers to food that is consumed without undergoing any thermal heating or cooking processes) concept in Fangchenggang is the preference for “raw food”, particularly raw fish slices. This preference is rooted in Fangchenggang's history, where the abundance of small and medium-sized rivers and rich water resources has significantly influenced consumption patterns. The combination of fresh raw fish slices with locally produced spices, including *Piper sarmentosum*, *Persicaria viscosa*, *Perilla frutescens* var. *purpurascens*, *Houttuynia cordata*, and *Ocimum basilicum*, showcases the distinctive culinary characteristics of the region. At present, raw fish slices have not only become a staple in daily meals but also play a crucial role in festive celebrations and hospitality. Serving raw fish to important guests embodies tradition and solemnity and acts as a means of communication, fostering emotional connections during social gatherings.

### Sourness

In Guangxi, there is a particular affinity for "sour food" throughout the region [75]. Although Fangchenggang leans more toward a preference for “freshness”, the level of sourness in their cuisine is slightly lower compared to other areas. Nevertheless, “sour food” remains a distinctive aspect of the local culinary culture.

Due to the underdeveloped economy and limited transportation, wild collecting is a time-consuming process, and in the humid and hot local climate, seasonal fruits, vegetables, and meats are prone to spoilage. To address these problems and ensure long-term consumption, the practice of pickling or fermenting food into sour varieties emerged. This preservation method allowed them to store and enjoy a variety of foods for an extended period. Examples of sour food in Fangchenggang include *Colocasia esculenta* (escaped populations), *Capsella bursa-pastoris*, *Phyllostachys heteroclada*, and *Pleioblastus amarus*. The specific selection of ingredients for pickling varies depending on the season and the availability of fresh produce.

Furthermore, in humid and hot climate, the Zhuang people engage in physically demanding work and often perspire heavily. Due to a relatively low level of stomach acid, consuming sour food can aid digestion, balance greasiness, and stimulate appetite [76]. As a result, aside from pickled sour food, they intentionally incorporate ingredients with sour flavors into their cuisine. For example, *Citrus limonia*, *Ananas comosus* (cultivated plants), *Passiflora edulis* (cultivated plants) is used to prepare dishes such as lemon duck, added to cold rice noodles, or used to create dipping sauces for boiled chicken or duck. These sour ingredients not only enhance the flavors of the dishes but also provide a refreshing and appetizing element to the overall culinary experience.

### Cold dishes

Living in mountainous areas, the Zhuang people prioritized the convenience of carrying and storing food during their labor activities, leading them to develop the practice of preparing food in a chilled or cold dietary culture. For instance, the leaves of *Musa balbisiana* and *Phyllostachys heteroclada* are commonly used to wrap glutinous rice with meat or beans. *P. heteroclada* is also used to steam rice, making it convenient for carrying. A dish called “qingtuan” made by mixing *Artemisia argyi* with sticky rice, can also be consumed as a cold dish, another type of rice cake, known as “Ciba” is prepared by mixing *Momordica cochinchinensis* fruit flesh with glutinous rice flour.

Compared to hard time in the past, these traditional delicacies are now celebrated during important festivals, sold as popular snacks at tourist attractions, and featured as main dishes in upscale restaurants. On the other hand, preference for light and cold dishes was also influenced by traditional medicine and healthcare. In Zhuang medicine, “cold dish” is considered a beneficial health practice, and regular consumption of cold dishes is advised.

## Conclusion

In summary, 163 WEPs and associated traditional knowledge used by Zhuang people were recorded. Multiple uses of these WEPs were analyzed, and the most culturally significant WEPs of the Zhuang people were identified by quantitative methods. From the historical development of wild edible plants consumption culture in Fangchenggang, it becomes evident that this cultural practice has been deeply influenced by the natural and social environments on which the Zhuang ethnic group relies for their long-term survival and prosperity. The specific living space and social interactions within their community have shaped a diverse and distinctive dietary tradition of consuming wild edible plants, which are notably characterized by “sour”, “fresh”, and “cool dishes” flavors. Moreover, they follow a health-oriented philosophy of “homology of medicine and food”, as evidenced by their longstanding customs of consuming Liáng chá and incorporating fresh wild vegetables and fruits into their diet.

Meanwhile, by exploring the functional and cultural transformation of these traditional delicacies within the Zhuang community, we also can gain valuable insights into the cultural changes in Fangchenggang. This includes shifts in production, lifestyle, and the dynamic interplay of different cultures in this emerging tourism city. Traditional cuisine has been reimagined, acquiring new cultural connotations and embracing its role as a medium for cultural exchange and expression.

In the future, wild vegetables and fruits with economic and medicinal potential can be further developed to serve as a source of income for local residents. The valuable traits of these wild edible plants (WEPs) can be preserved and enhanced through cross-breeding to create new varieties that cater to market demands. To ensure the safe consumption of wild edible plants, it is essential to conduct rigorous analysis, testing, and safety evaluations on those with high cultural food significance index (CFSI) values. Meanwhile, attention should also be paid to the protection of wild edible plants and associated traditional knowledge, this will ensure that this valuable knowledge is not lost to future generations.

## Data Availability

All data generated or analyzed during this study was included in this published article (along with the supplementary files).

## Abbreviations

AI: Availability index
ASEAN: Association of Southeast Asian Nations
CFSI: Cultural food significance index
FMRI: Food-medicinal role index
FUI: Frequency of utilization index
MFFI: Multifunctional food use index
PUI: Parts used index
QI: Frequency of quotation index
TSAI: Taste score appreciation index
WEP: Wild edible plant
Wv: Wild vegetables
Wf: Wild fruits
Nu: Nuts
Sp: Spices
Ts: Tea substitutes
Lb: Liquor brewing
Of: Oils and fats
Sn: Snack
Fd: Food dyeing
LC: Least concern
EN: Endangered
Vu: Vulnerable
